# Relative Residence Time Can Account for Half of the Anatomical Variation in Fatty Streak Prevalence Within the Right Coronary Artery

**DOI:** 10.1007/s10439-024-03607-9

**Published:** 2024-09-17

**Authors:** Pratik Kandangwa, Kevin Cheng, Miten Patel, Spencer J. Sherwin, Ranil de Silva, Peter D. Weinberg

**Affiliations:** 1 Department of Bioengineering, Imperial College London, London, SW7 2AZ UK; 2 Department of Aeronautics, Imperial College London, London, SW7 2AZ UK; 3 National Heart and Lung Institute, Imperial College London, London, SW3 6LY UK; 4 Royal Brompton Hospital, Sydney Street, London, SW3 6NP UK

**Keywords:** Coronary artery disease, Hemodynamics, Computational fluid dynamics, Endothelial cell activation potential, Pathobiological determinants of atherosclerosis in youth

## Abstract

**Purpose:**

The patchy anatomical distribution of atherosclerosis has been attributed to variation in haemodynamic wall shear stress (WSS). The consensus is that low WSS and a high Oscillatory Shear Index (OSI) trigger the disease. We found that atherosclerosis at aortic branch sites correlates threefold better with transverse WSS (transWSS), a metric which quantifies multidirectional near-wall flow. Coronary artery disease has greater clinical significance than aortic disease but computation of WSS metrics is complicated by the substantial vessel motion occurring during each cardiac cycle. Here we present the first comparison of the distribution of atherosclerosis with WSS metrics computed for moving coronary arteries.

**Methods:**

Maps of WSS metrics were computed using dynamic geometries reconstructed from angiograms of ten non-stenosed human right coronary arteries (RCAs). They were compared with maps of fatty streak prevalence derived from a previous study of 1852 RCAs.

**Results:**

Time average WSS (TAWSS), OSI, transWSS and the cross-flow index (CFI), a non-dimensional form of the transWSS, gave non-significant or significant but low spatial correlations with lesion prevalence. The highest correlation coefficient (0.71) was for the relative residence time (RRT), a metric that decreases with TAWSS and increases with OSI. The coefficient was not changed if RRT was calculated using CFI, which captures multidirectional WSS only, rather than OSI, which encompasses both multidirectional and oscillatory WSS.

**Conclusion:**

Contrary to our earlier findings in the aorta, low WSS in combination with highly multidirectional flow correlates best with lesion location in the RCA, explaining approximately half of its anatomical variation.

**Supplementary Information:**

The online version contains supplementary material available at 10.1007/s10439-024-03607-9.

## Introduction

Atherosclerosis develops preferentially at certain well-defined sites within the vascular system [1–3]. This patchy distribution implies the existence of local risk factors, the identification of which could lead to new prognostic methods and therapeutic strategies. A widely investigated possibility is that lesion development depends on some feature of haemodynamic wall shear stress (WSS).

The current consensus is that disease is initiated in regions experiencing low and oscillatory WSS [4, 5]. A recent review, however, showed that all studies which conducted a pointwise statistical comparison of lesion prevalence and computed WSS contradict this hypothesis [6]. A new metric, the transverse wall shear stress (transWSS) [7], correlated 3-fold better than time averaged wall shear stress (TAWSS) or the oscillatory shear index (OSI) [8] with lesion prevalence around branch mouths in the rabbit descending thoracic aorta [9]. TransWSS encapsulates multidirectional flow by averaging over the cardiac cycle those components of the instantaneous WSS vectors that are perpendicular to the mean WSS vector.

Atherosclerosis of the coronary arteries accounts for around one in six deaths globally [10]. Consequently, WSS metrics such as the TAWSS, OSI, WSS Gradient (WSSG) [11] and Relative Residence Time (RRT [12]) have been extensively studied in coronary CFD models [e.g. 8, 13–16]. More recently, transWSS has also been examined. Pedrigi et al. [17] found a spatial correlation between transWSS and advanced lesions induced by a shear-modifying stent in hypercholesterolaemic minipigs. On the other hand, a prospective study of human coronary arteries [18] found that transWSS was not significantly correlated with the change in total plaque, fibrous tissue or fatty-fibrous tissue over time, and a similar finding was obtained for porcine coronary arteries (Fig. 1D in [19]).

These studies computed flow in static geometries. In vivo, coronary arteries undergo bending and translation during the cardiac cycle. Several studies have assessed the influence of coronary motion on WSS metrics [20–27]. There was little effect on TAWSS, except in one study [24] of a highly stenosed vessel. Many studies showed increased fluctuation of local WSS vectors with time and an increased OSI. Kandangwa et al. [26] recently demonstrated for the right coronary artery (RCA) that transWSS was altered even more than the OSI. Fogell et al. [27] subsequently confirmed a large influence of bending on transWSS in all three coronaries. Hence coronary motion should be taken into account when attempting to relate the development of atherosclerosis to WSS metrics other than TAWSS.

Here, for the first time, we determine spatial correlations of early lesion prevalence with WSS metrics computed for a moving coronary artery. Lesion prevalence maps were re-derived from the Pathobiological Determinants of Atherosclerosis in Youth (PDAY) study [28]. The computational model incorporated side branches, which are necessary to maintain a physiological flow rate on proceeding distally along the vessel [26]; they have been ignored in many previous studies so simulations were also conducted without branches, to examine their effect. Additionally, because flow could not be measured in the subjects whose coronary geometries were used, three previously published inflow waveforms were employed to examine sensitivity to this boundary condition. Simulations were also run for static geometries, in order to assess the effects of coronary motion on correlations with lesion prevalence.

## Methods

### Angiography and 3-D Reconstruction of Angiograms

Ten subjects were selected from patients undergoing a routine investigation at the Royal Brompton Hospital that included invasive coronary angiography. Patients were excluded if they had a > 30% stenosis at any point in the RCA, as visually judged from their angiograms, and equal numbers of men and women were chosen. The study was performed in accordance with the ethical standards laid down in the 1964 Declaration of Helsinki and its later amendments and received independent approved from the Health Research Authority (22/HRA/4349; IRAS project ID: 318558); in that approval, patient consent for use of pseudo-anonymised data obtained as part of routine clinical care was not required.

Angiograms were acquired at 15 frames/s and from two views (angle separation ≥ 30°) after intracoronary administration of isosorbide dinitrate (300–500 μg). 3-D geometries were reconstructed from the origin of the RCA to the posterior descending artery at 0.07-s intervals throughout the cardiac cycle using CAAS Intravascular Software (Pie Medical Imaging BV, the Netherlands), previously validated against a combination of angiography and intravascular ultrasound [29]. (A diagrammatic summary of the workflow is given in Supplementary Material Fig. S1.) An elliptical cross-section was imposed. All visible branches (n = 2–5) were reconstructed for a length of approximately 1.5 cm. The software only allowed reconstruction of one branch of the main vessel. Each branch was therefore individually reconstructed along with the main vessel; diameters and circumferential and longitudinal positions were subsequently used to add all branches, idealised as cylindrical tubes, to a single main vessel, using SOLIDWORKS (Dassault Systèmes, France).

### CFD Modelling

Time-dependent flow simulations were carried out using STAR-CCM+ (Siemens Digital Industries Software Ltd., Germany). Details of the CFD model, and how wall motion was applied are described elsewhere [26]. The elliptical cross section was kept constant at each lengthwise location throughout the cardiac cycle. (The geometry at the start of systole was used for the static simulations.) Blood was modelled as a laminar incompressible Newtonian fluid with a density of 1044 kg/m^3^ and a dynamic viscosity of 4.043 × 10^−3^ Pas.

**Fig. 1:**
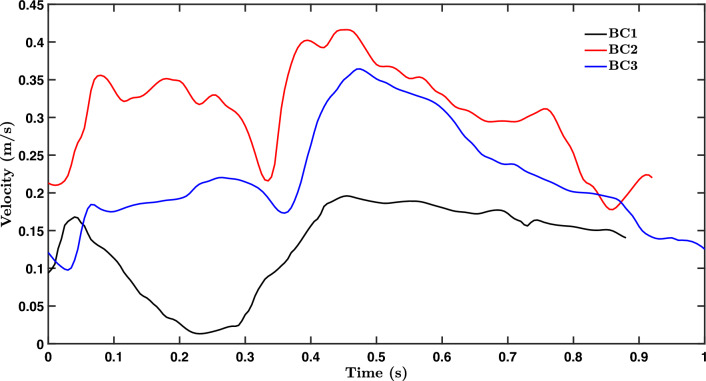
Variation in inlet velocity over the cardiac cycle, measured in three previous studies

Three published RCA inlet velocity waveforms (BC1 [30], BC2 [31] and BC3 [32]; Fig. 1), all acquired using a Doppler flow wire, were used. BC2 was used when assessing effects of coronary motion. A parabolic inlet velocity profile was imposed, with the measured velocity being its maximum. A mass flow rate boundary condition was used for the side branches, with the flow ratio proportional to diameter raised to the empirically determined power of 2.27 [33–35]. All simulations were carried out with inlet and outlet extensions of length equal to 3 diameters.

WSS vectors were used to calculate TAWSS, OSI, transWSS, and the Cross Flow Index (CFI—a dimensionless version of the transWSS [36]). The endothelial cell activation potential (ECAP) [37] and RRT [12] were obtained by combining these, and analogous metrics—the ECAP_CFI_ and RRT_CFI_—were obtained by using the CFI instead of the OSI in their calculation. Equations for these metrics are given in Supplementary Material Table S1.

The 3-D representations of WSS metrics produced by STAR-CCM+ were unwrapped, converting them to 2-D rectangular maps [26]. The cut was made along the outer wall (i.e. the wall furthest from the surface of the heart), consistent with the cuts made in the PDAY lesion prevalence study.

### Disease Prevalence Maps

Digital maps of the prevalence of fatty streaks were re-derived from previously published images. The original PDAY study was designed to examine effects of risk factors on atherosclerosis in relatively young subjects. Protocols are given elsewhere [28, 38, 39]. Briefly, subjects were aged 15–34 years and died from suicide, homicide or accident between 1987 and 1994. Fifty-two percent of cases were black and 76% were men; vessels were collected within 72 h of injury and 48 h of death, fixed in 10% formalin for 48 h, stained with Sudan IV and graded automatically after digitisation of 35-mm transparencies. The figures we used to derive new digital maps were those which averaged fatty streak prevalence for the first 6 cm of the RCA separately for men and women and for four 5-year age groups [28].

Online versions of the original figures were processed using custom Matlab code to convert each banded isopleth map into a single matrix. First, a custom colour map consisting of the six colours seen in the published figures was constructed. Using this colour map the images were then converted into three matrices with red, green and blue (RGB) pixel values. Finally, the RGB matrices were transformed into a single matrix with index ranging from 0 to 5.

In the PDAY study, arteries were excised and then fixed in formalin. They would therefore have shrunk compared to the dimensions obtained from the in vivo angiographic images used to create the CFD models. A number of studies have measured such shrinkage (e.g. [17, 40]). To compensate, the prevalence maps were stretched by 33%, increasing their effective length to 8 cm.

### Statistics

Histograms were computed to determine whether different inflow waveforms or the removal of side branches altered the magnitude and frequency distribution of values obtained for each WSS metric.

Spearman’s rank correlation coefficient (ρ) was computed between average maps of WSS metrics and lesion prevalence in order to quantify similarities without assuming a linear relation. To assess statistical significance whilst avoiding problems caused by autocorrelation within the maps [41], confidence intervals (CIs) were computed for the correlation coefficients by bootstrapping [41–44], as outlined in Supplementary Material Appendix A. The correlation was deemed significant if the CI excluded 0. The same statistical approach was used when comparing WSS maps with and without branches, and WSS maps using different inlet velocity waveforms.

## Results

### Subject Characteristics

The ten subjects were aged 44–79 years (mean 64, interquartile range 54–68). Co-morbidities included type 2 diabetes mellitus (n = 2), hypertension (n = 3) and previous smoking history (n = 3). A summary of geometrical features and flow parameters for each subject is given in Supplementary Material Table S2.

### Effect of Inflow Waveform on RCA Haemodynamics

**Fig. 2:**
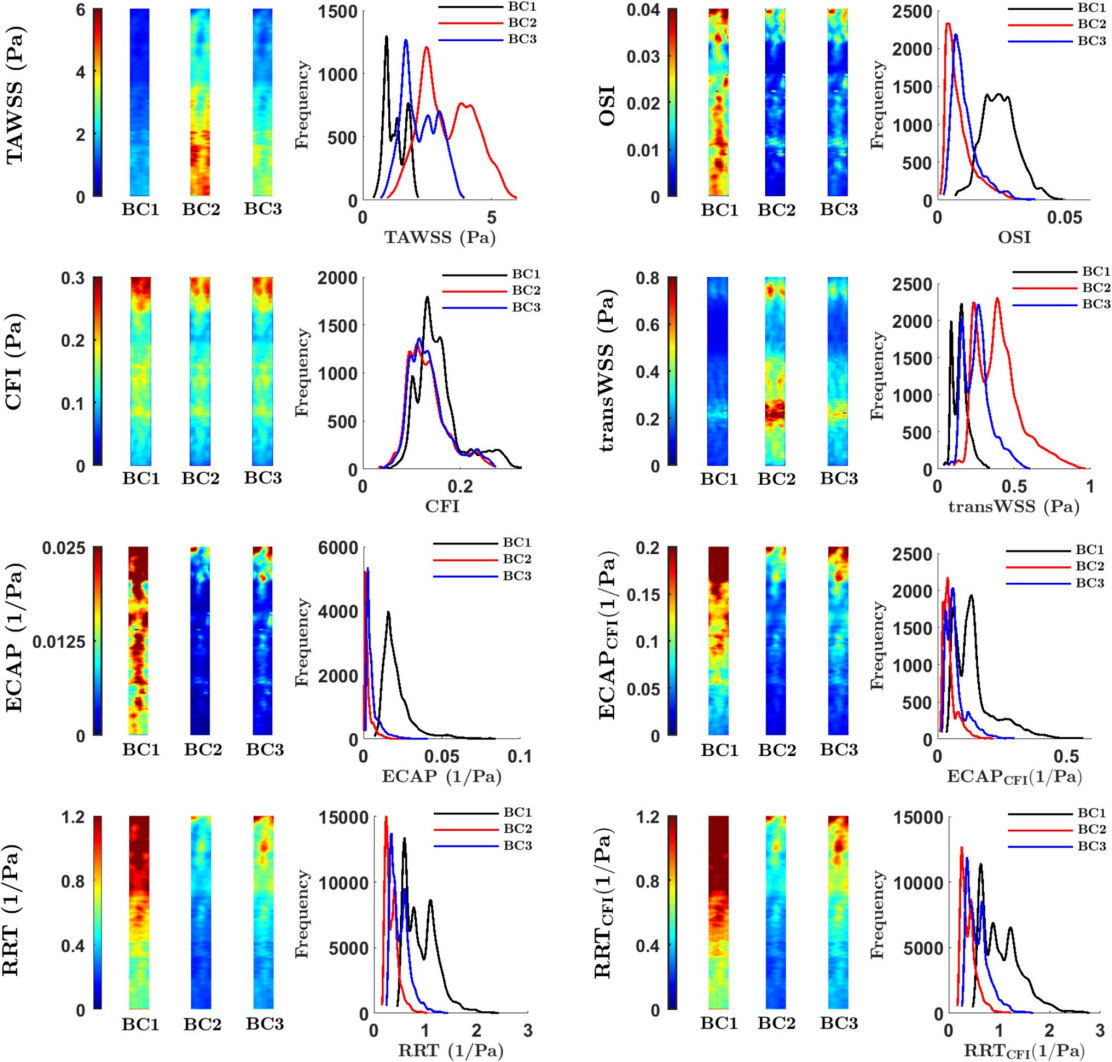
Maps showing WSS metrics averaged across subjects, and corresponding frequency histograms, computed for the three inlet waveforms BC1–BC3. The proximal end of the RCA is at the top of each map. Geometries did not include branches

The three inflow waveforms had different average velocities; TAWSS and transWSS, which are dimensional, therefore showed corresponding differences in average magnitude. Nevertheless, it is evident from the maps that patterns of TAWSS and transWSS did not change with inflow waveform, and the overall shape of the frequency histogram also remained the same for these two metrics, despite its shift along the abscissa (Fig. 2; means and standard deviations of the values for each map are given in Supplementary Material Table S3).

These observations are quantified by the Spearman’s ranked correlation coefficients and 95% CIs: high ρ values (all ≥ 0.95), coupled with a high lower confidence bound (all ≥ 0.85), show that the patterns of TAWSS and transWSS metrics remained consistent across waveforms (Table 1).

**Table 1:** Spearman’s rank correlation coefficients (ρ) and bootstrapped confidence intervals (CI) obtained by pointwise comparison of WSS maps computed using three different inflow waveforms (BC1–BC3)

WSS metric	BC1 vs BC2	BC1 vs BC3	BC2 vs BC3
	ρ [CI]	ρ [CI]	ρ [CI]
TAWSS	0.98 [0.95, 0.99]	0.99 [0.98, 0.99]	0.99 [0.98, 1.00]
OSI	0.55 [0.51, 0.73]	0.71 [0.63, 0.84]	0.95 [0.89, 0.98]
CFI	0.95 [0.91, 0.96]	0.97 [0.93, 0.97]	0.99 [0.98, 0.99]
transWSS	0.95 [0.85, 0.96]	0.97 [0.92, 0.98]	0.99 [0.97, 0.99]
ECAP	0.90 [0.72, 0.92]	0.95 [0.84, 0.96]	0.98 [0.94, 0.98]
ECAP_CFI_	0.96 [0.94, 0.97]	0.97 [0.96, 0.98]	0.99 [0.99, 1.00]
RRT	0.98 [0.96, 0.98]	0.99 [0.98, 0.99]	1.00 [1.00, 1.00]
RRT_CFI_	0.97 [0.96, 0.98]	0.99 [0.98, 0.99]	1.00 [1.00, 1.00]

The same was broadly true for the CFI: the maps for the three waveforms were visually indistinguishable and ρ values were ≥ 0.95, with lower confidence bounds ≥ 0.91, although the frequency distribution for BC1 was shifted right by approximately 25% despite the metric being dimensionless, and had a higher peak value than the distributions for BC2 and BC3 (Fig. 2 and Table 1).

OSI, on the other hand, had a markedly different pattern when comparing inflow waveform BC1 with the other two boundary conditions: a streak of high values occurred in the distal half of the inner curvature of the vessel only with BC1, leading to ρ values of 0.55 and 0.71 with BC2 and BC3, respectively. (The corresponding upper confidence bounds were 0.73 and 0.84.) The frequency distribution was shifted right by a factor of three and blunted (Fig. 2 and Table 1).

The coefficients for ECAP and ECAP_CFI_, varied between 0.90 and 0.97 when BC1 was compared with BC2 or BC3, the lower values being for the formulation based on OSI, but were 0.98 and 0.99 when BC2 was compared with BC3. The coefficients for RRT and RRT_CF_ were uniformly high (all ≥ 0.97). Thus ECAP and RRT seem to have a different sensitivity to fluctuations in OSI.

### Effect of Side Branches on RCA Haemodynamics

**Fig. 3:**
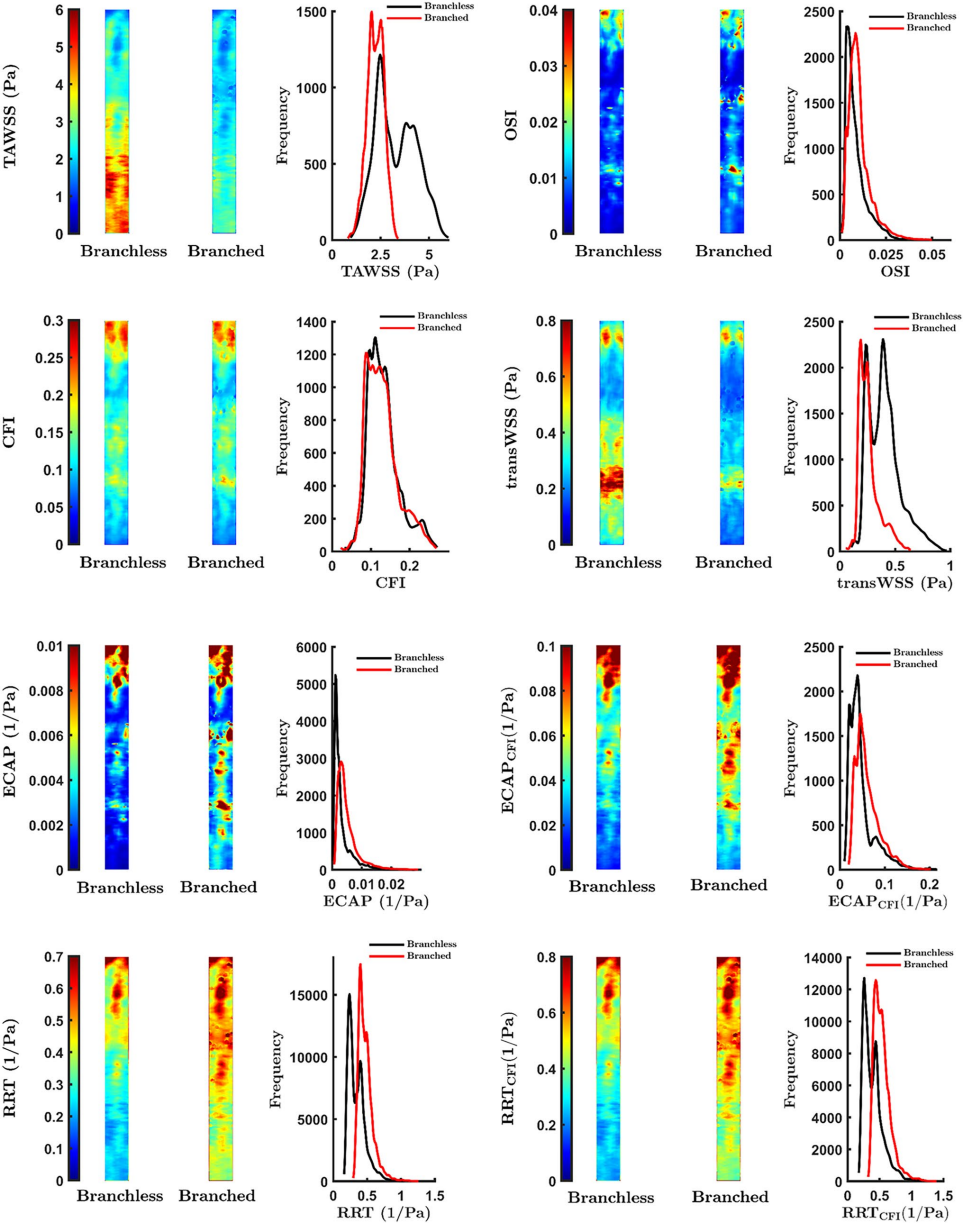
Maps showing WSS metrics, averaged across subjects, and corresponding frequency histograms for branched and branchless configurations. The proximal end of the RCA is at the top of each map

Branches affected both the magnitude and pattern of the dimensional metrics, TAWSS and transWSS. The histograms show that these metrics lost their high values when branches were incorporated; that is consistent with the maps, which show that adding branches led to a progressive reduction in magnitudes on proceeding down the vessel (Fig. 3; means and standard deviations of the values for each map are given in Supplementary Material Table S4). Similarly, the dimensional ECAP, ECAP_CFI_, RRT and RRT_CFI_ lost their lowest values. (These metrics increase when TAWSS decreases.) Local effects of branches were not particularly apparent in the average maps, presumably reflecting variability in branch location between subjects. Values of ρ between branched and branchless cases (Table 2) were 0.85 for TAWSS and 0.83 for transWSS. The pattern and magnitude of the CFI were almost entirely unaffected by including branches but OSI did show some difference (ρ = 0.83). Coefficients for ECAP, ECAP_CFI_, RRT and RRT_CFI_ were similar to, or slightly greater than, the value for OSI (0.83–0.89).

**Table 2:** Spearman’s rank correlation coefficient (ρ) and bootstrapped confidence intervals (CI) obtained by pointwise comparison of WSS maps computed with or without branches

WSS metric	Branched vs no branches
	ρ [CI]
TAWSS	0.85 [0.65, 0.93]
OSI	0.83 [0.59, 0.93]
CFI	0.97 [0.92, 0.97]
transWSS	0.83 [0.69, 0.89]
ECAP	0.86 [0.69, 0.92]
ECAP_CFI_	0.89 [0.81, 0.93]
RRT	0.83 [0.71, 0.88]
RRT_CFI_	0.84 [0.73, 0.89]

### Correlation of WSS Metrics with Fatty Streak Prevalence

**Fig. 4:**
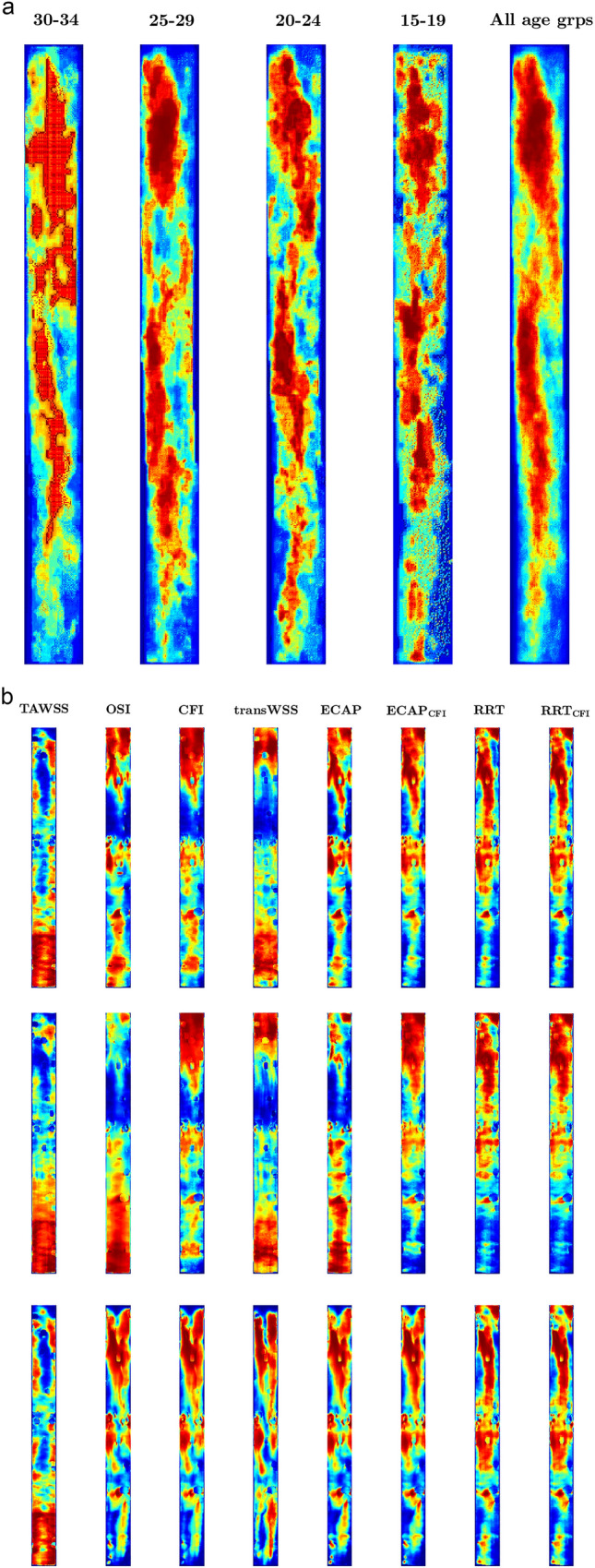
(a) Maps showing fatty streak prevalence averaged across subjects of the PDAY study for four individual age ranges (values in years) and for all ages combined. (b) Maps showing WSS metrics averaged across subjects for dynamic coronary geometries with inflow waveforms BC2 (top row) and BC1 (middle row), and for static coronary geometries with inflow waveform BC2 (bottom row). Colour coding shows rank within each map (e.g. dark blue = lowest ranked value). The proximal end of the RCA is at the top of each map

Patterns of lesion prevalence computed separately for men and women (Supplementary Material Fig. S2) were similar to one other, although prevalence was lower in women, as expected. The data for men and women were therefore combined; maps were constructed for the original age groups, but were also averaged across age groups (Fig. 4a). Colour coding by rank rather than absolute value was used for all maps, to facilitate comparisons between them and for consistency with the rank correlation coefficients.

Lesion prevalence did not show any striking change in pattern with age. (It increased, but that cannot be seen with the rank colour scale.) The fundamental distribution appeared to be helical (Supplementary Material Fig. S3), as previously shown for the left coronary artery [3]. The helix rotated clockwise.

For the initial comparison with maps of lesion prevalence, maps of WSS metrics were computed using branched geometries, inlet velocity waveform BC2 and dynamic geometries. To allow assessment of individual variation under these conditions, maps for individual subjects are shown in Supplementary Material Figs. S4–S11, as are maps of the coefficient of variance (SD/mean; Supplementary Material Fig. S12). The modal value of the coefficient of variation was < 0.5 for TAWSS, transWSS, CFI, RRT and RRT_CFI_ but 0.63 for ECAP_CFI_ and > 0.8 for OSI and ECAP.

Maps of TAWSS, OSI, CFI and transWSS (Fig. 4b top row) did not show much resemblance to the maps of fatty streaks (Fig. 4a). For example, OSI and CFI had low values towards the end of the first third of the vessel, and TAWSS and transWSS had high values at the distal end, both trends being sharply demarcated and involving most of the circumference, yet both regions had intermediate values of lesion prevalence with a highly nonuniform circumferential distribution. This observation was confirmed by the correlation coefficients in Table 3: the coefficient for transWSS did not reach statistical significance and although coefficients for the other basic metrics did, even the highest value of ρ, which was for CFI, was only 0.44. ECAP and RRT, and the analogous metrics calculated with CFI rather than OSI, all had a similar pattern, and it correlated much better with the map of lesion prevalence. The highest correlations were with RRT and RRT_CFI_, which give values of ρ of 0.61–0.71.

**Table 3:** Spearman’s rank correlation coefficients (ρ) and bootstrapped confidence intervals (CI) obtained by pointwise comparison of maps of lesion prevalence in different age groups (values in years), or averaged across age groups, with maps of eight WSS metrics

Age group	30–34	25–29	20–24	15–19	All age groups
WSS metric	ρ [CI]	ρ [CI]	ρ [CI]	ρ [CI]	ρ [CI]
TAWSS	− 0.19 [− 0.32, − 0.054]	− 0.14 [− 0.31, − 0.01]	− 0.16 [− 0.31, − 0.036]	− 0.21 [− 0.35, − 0.094]	− 0.23 [− 0.38, − 0.11]
OSI	0.11 [− 0.11, 0.28]	0.23 [0.049, 0.35]	0.31 [0.11, 0.41]	0.28 [0.11, 0.37]	0.25 [0.027, 0.39]
CFI	0.28 [0.03, 0.41]	0.39 [0.13, 0.46]	0.44 [0.19, 0.51]	0.39 [0.14, 0.44]	0.42 [0.11, 0.51]
transWSS	− 0.12 [− 0.26, 0.18]	0.028 [− 0.12, 0.26]	0.068 [− 0.048, 0.26]	− 0.006 [− 0.13, 0.21]	− 0.028 [− 0.17, 0.24]
ECAP	0.25 [0.03, 0.35]	0.34 [0.18, 0.41]	0.39 [0.23, 0.47]	0.39 [0.25, 0.44]	0.38 [0.19, 0.46]
ECAP_CFI_	0.42 [0.25, 0.48]	0.47 [0.34, 0.52]	0.51 [0.36, 0.58]	0.49 [0.36, 0.53]	0.55 [0.38, 0.58]
RRT	0.62 [0.44, 0.63]	0.61 [0.42, 0.62]	0.64 [0.41, 0.65]	0.63 [0.44, 0.64]	0.71 [0.51, 0.72]
RRT_CFI_	0.61 [0.45, 0.64]	0.61 [0.41, 0.63]	0.63 [0.44, 0.65]	0.64 [0.44, 0.65]	0.69 [0.53, 0.71]

**Table 4:** Spearman’s rank correlation coefficients (ρ) obtained by pointwise comparison of the map of lesion prevalence averaged across age groups with maps of eight WSS metrics, calculated for inflow condition BC1 or for BC2 combined with a static geometry

WSS metric	BC2	BC1	Static
TAWSS	− 0.23	− 0.15	− 0.20
OSI	0.25	− 0.021	0.45
CFI	0.42	0.39	0.51
transWSS	− 0.028	− 0.013	0.41
ECAP	0.38	0.17	0.46
ECAP_CFI_	0.55	0.51	0.53
RRT	0.71	0.62	0.64
RRT_CFI_	0.69	0.61	0.64

The coefficients for BC2 with a dynamic geometry are repeated from the last column of Table 3 for comparison

The maps showed a patchy distribution of OSI, CFI, ECAP and RRT approximately halfway along the segment that appears to reflect the variable location of major branches in this region, since shapes resembling branch ostia are visible (Supplementary Material Fig. S3). Such shapes were not seen in the lesion prevalence maps, presumably because of the much larger sample size.

### Effect of Inlet Waveform and Coronary Motion on Correlations

Simulations were also run with inlet waveform BC1, to determine whether its lower velocities would alter the correlations. (The simulations were similar to those shown in Fig. 2, but branches were included.) Additionally, BC2 was used with static rather than dynamic geometries to additionally determine the influence of arterial movement. Spearman’s rank correlation coefficients between the lesion prevalence map for all ages combined (Fig. 4a) and maps of WSS metrics obtained for BC1 (Fig. 4b middle row) were in the same order as the equivalent coefficients for BC2 except that the coefficient for OSI dropped from third lowest (ρ = 0.25) to second lowest (ρ = − 0.021; Table 4). That is consistent with the influence switching from BC2 to BC1 had on the frequency distribution of WSS metrics, where OSI was the most affected (Fig. 2).

Spearman’s rank correlation coefficients between maps of WSS metrics obtained with dynamic geometries (Fig.﻿ 4b top row) and the equivalent maps obtained with static geometries (Fig. 4b bottom row) were: TAWSS, 0.95; OSI, 0.45; CFI, 0.55; transWSS, 0.22; ECAP, 0.67; ECAP_CFI_ 0.79; RRT, 0.92; and RRT_CFI_,0.91. This is consistent with our previous studies showing that right coronary motion had little effect on TAWSS, more on OSI and even more on transWSS [26]. The similar coefficients for OSI and CFI are again consistent with the former being determined largely by multidirectional flow in this vessel. The higher values for ECAP, ECAP_CFI_, RRT and RRT_CFI_ are consistent with the fact that TAWSS is included in their definition.

The switch to static geometries evened out the rank correlation coefficients between the lesion prevalence map and WSS maps (Table 4). The low coefficients obtained in dynamic geometries for OSI, CFI and particularly transWSS were increased, the intermediate values previously obtained for ECAP and ECAP_CFI_ remained largely unchanged, and the high values obtained for RRT and RRT_CFI_ were reduced. Excluding TAWSS (which retained the same distribution and hence the same, low correlation coefficient), the range of coefficients reduced more than three-fold, from − 0.028–0.71 under dynamic conditions to 0.41–0.64 under static conditions.

## Discussion

This study used computational methods to simulate flow in reconstructions of the geometry of ten human RCAs with minimal disease. A finite volume approach in STAR CCM+ was combined with the morphing solver to model vessel motion. Assumptions were: continuum fluid mechanics, an incompressible medium with Newtonian rheology, laminar flow, and elliptical vessel cross sections that were constant over the cardiac cycle at each location. Vessel translation, changes in curvature (in-plane bending) and changes in torsion (out-of-plane bending) were incorporated. Branches were also incorporated. Our previous study [26] showed little effect on shear metrics of the wall moving past the fluid, rather than fluid moving past the wall; the former effect was included here but was not quantified separately. Twisting of the vessel (as might be seen in a regular cylinder without the introduction of curvature or torsion) might add to such effects, but it could not be imaged and hence was not included in either study.

Since subject-specific inflow waveforms could not be obtained, the sensitivity of WSS metrics to this boundary condition was examined by imposing three previously published waveforms. The anatomical pattern of all but one of the metrics was effectively unchanged between the three, although absolute magnitudes for the dimensional metrics did vary. This is consistent with our previous finding that the effect of wall motion far outweighs the effect of flow pulsatility [26].

**Fig. 5:**
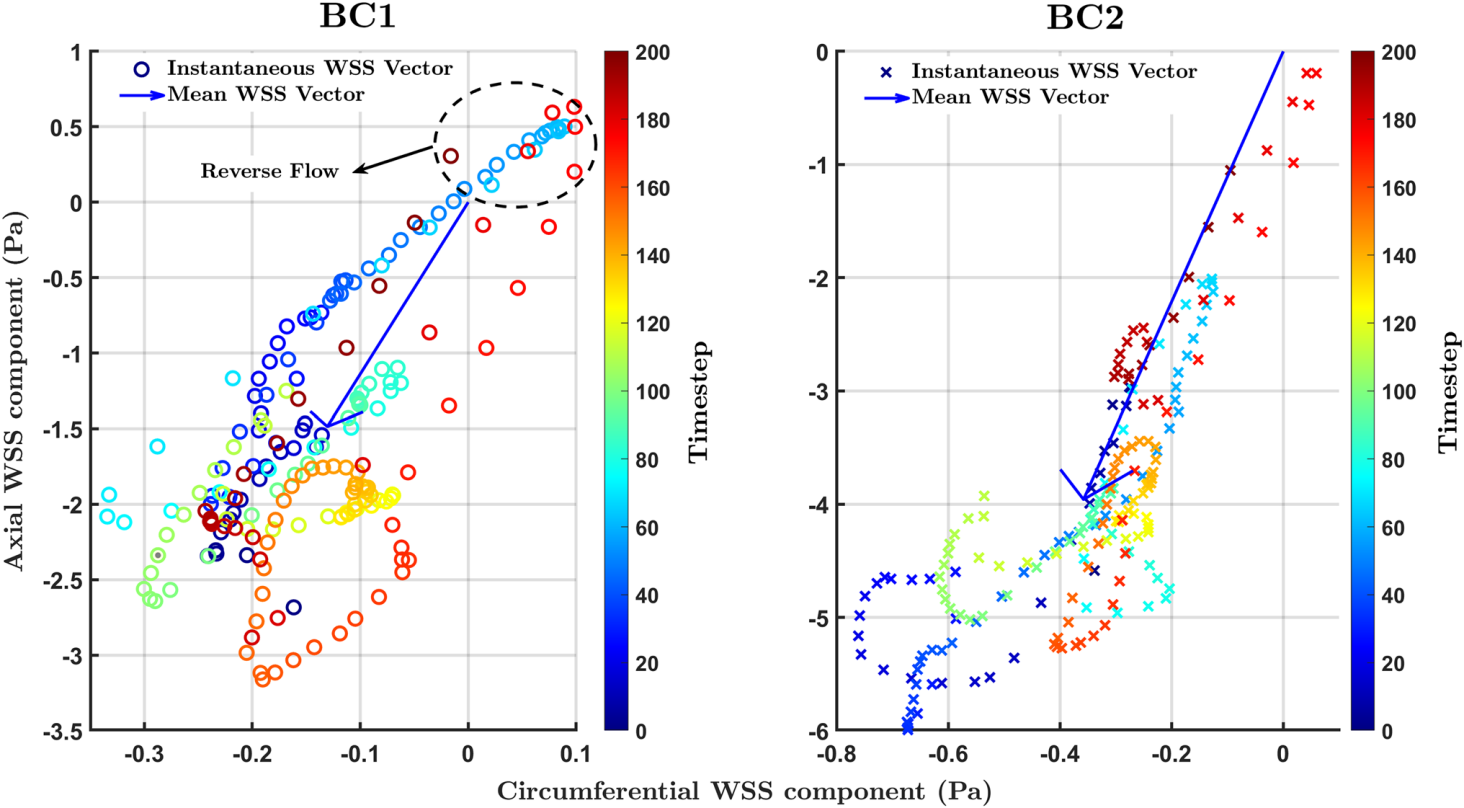
Polar plots tracking the direction and magnitude of the instantaneous WSS vector over the cardiac cycle at a single location on the wall. Simulations used inflow waveform BC1 (left) or BC2 (right). Each point represents the position of the tip of one instantaneous vector with its origin at 0,0. The X and Y axes represent WSS components oriented along the circumference and axis of the vessel, respectively, with proximal-to-distal flow producing negative axial values. Colour coding shows time throughout the cardiac cycle. The blue arrow is the mean vector. BC1 produces more instantaneous vectors than BC2 that are in the opposite direction to the mean vector. (Note the different scales.)

The exception was OSI; it did show a different pattern when using waveform BC1, which had substantially lower velocities than BC2 and BC3. Although the lower cross-sectionally averaged velocity was forward directed throughout the cardiac cycle, it appears to have led to reverse near-wall flow, which was not seen with the other inlet waveforms (Fig. 5). The OSI, which reflects both back-and-forwards and multidirectional WSS, tended towards the pattern of CFI for the inflow waveforms with higher velocities: Spearman’s rank correlation coefficient between OSI and CFI increased from 0.24 for BC1 to 0.83 for BC3 and 0.93 for BC2. This shows that at higher forward velocities, OSI primarily reflected cross flow rather than purely reversing flow. We conclude that patterns of shear metrics are insensitive to inflow waveform with the exception of OSI, which can alter if velocities become sufficiently low.

Side branches have been omitted in some earlier simulations of RCA flow, including our own. We therefore also examined the effect of branches on WSS metrics. Although only one of the inflow waveforms was used, that was sufficient to show that omitting branches can artefactually increase mean flow on proceeding distally along the vessel. Dimensional metrics (TAWSS and transWSS) therefore showed changes in both magnitude and pattern. OSI also changed, albeit to a lesser extent. In some studies [23], RCA taper has been removed to adjust for missing branches but it is not clear that the physiological degree of taper is determined solely by a need to compensate WSS for branch outflow. We conclude that it is important to include RCA branches when considering dimensional WSS metrics.

Our main aim was to assess potential haemodynamic triggers of atherosclerosis. Maps of WSS metrics were compared with maps of fatty streak prevalence using point-by-point correlations. The following discussion is based on the map of lesion prevalence averaged across age groups, but the conclusions are unaffected by using the map for any single age group.

TAWSS was generally low along the inner curvature, except at the distal end of the vessel, and lesion prevalence was also high at some locations along the inner curvature. Visual assessment might therefore have suggested an association, but the correlation coefficient was only − 0.23. The percentage of the spatial variation in lesion prevalence that is explained by TAWSS, which is given by the square of the correlation coefficient, is therefore around 5%. (Conventionally, this coefficient of determination is computed by squaring Pearson’s correlation coefficient; Spearman’s rank correlation coefficient, used here, is Pearson’s correlation coefficient applied to ranks of values rather than to the values themselves so, more rigorously, the coefficient of determination obtained by squaring it is the explained fraction of variance in ranked values rather than absolute values.)

The map of OSI also visually resembled the lesion prevalence map, especially in the distal half of the vessel, but the correlation coefficient was similarly low at 0.25. TransWSS did not give a significant correlation but CFI, which is the non-dimensional form of transWSS, gave a value of 0.41.

The ECAP performed nearly as well as the CFI (ρ = 0.38), and the RRT performed substantially better (ρ = 0.71). Both metrics combine TAWSS and OSI so that they increase with OSI and decrease with TAWSS. (We used a dimensional form of the RRT—i.e., one not normalised by RRT at a defined site—since that makes no difference when constructing maps of ranks or computing rank correlation coefficients. It is equivalent to the reciprocal of the magnitude of the cycle-average of the instantaneous WSS vectors and depends non-linearly on OSI, whereas ECAP depends linearly on it.)

Substituting CFI for OSI in the formulation of these metrics increased the correlation coefficient to 0.55 for ECAP_CFI_, while the coefficient for RRT_CFI_ was essentially unchanged at 0.69. Thus RRT and RRT_CFI_ explain approximately half the variation in ranked lesion prevalence across the maps. These results suggest a propensity for disease occurrence in regions characterized by low TAWSS combined with high OSI or CFI. It is the multidirectional nature of the near-wall flow rather than its back-and-forth motion that largely determines the OSI. Supplementary Material Appendix B discusses whether local values of TAWSS and multidirectionalilty could be predicted simply from geometric features of the RCA.

The order of the correlation coefficients between WSS metrics and lesion prevalence was little altered by the choice of inflow boundary condition; only OSI changed position, and by only one place. When an equivalent analysis was conducted using static rather than dynamic geometries, the rank correlation coefficients between WSS maps and lesion prevalence maps evened out, so that it became hard to determine which WSS metric correlated best with disease.

Although the low magnitude we obtained for the inverse correlation between TAWSS and lesion prevalence is controversial, three previous studies [15, 45, 46] calculating pointwise correlations in human coronary arteries have also failed to find a consistent, significant association. (These studies used intimal thickness or wall thickness as a surrogate for lesion severity or prevalence, which may introduce inaccuracies; for example, intimal cushions may be adaptive responses of the wall.)

Many previous studies [e.g. 45, 47–49] did show an inverse relation between wall thickness or plaque progression and instantaneous or time average WSS in human coronaries, but they averaged the biological and mechanical measurements in lengthwise segments of the vessel or in sectors around its circumference, rather than using a pointwise comparison. To investigate whether such averaging could account for the apparent discrepancy with the earlier studies, we averaged our own data. When lesion prevalence and TAWSS were averaged around the entire circumference in each of sixteen lengthwise segments of the vessel and then compared with each other, the absolute magnitude of the correlation coefficient became larger, changing from − 0.23 to − 0.59. Similarly, when RRT was averaged in each of 16 circumferential sectors running along the entire length of the vessel, the coefficient increased from 0.71 to 0.96. Although not all metrics were similarly affected, these figures show that correlations can be increased by averaging. In the past, this effect has been regarded as beneficial because it increased the negative correlation of disease with WSS [45]; we suggest that the raised significance is artefactual.

Kurtcuoglu et al. [50] showed for the right coronary artery that TAWSS had a higher sensitivity for the prediction of raised plaque location than did the WSSG, OSI or RRT, although it did not have the highest positive predictive value. The order of the sensitivities of the metrics was different to the order of the correlations found in the present study but the range of sensitivities across the four metrics was narrow (59–73%), as might be expected for a static geometry, and branches do not appear to have been included. Alternatively, there may be differences in the haemodynamic factors predisposing to fatty streaks, studied here, and raised lesions, studied by Kurtcuoglu et al., although the prevalence maps for the two types of lesion in the PDAY study of the RCA [28] are visually indistinguishable.

Previous investigations have found different relations in arteries other than the coronaries, such as our own study of transWSS and disease in the aorta [10]. Broadly, there are three possible explanations for such discrepancies: (i) haemodynamic metrics are not causally related to atherogenesis and the correlations are spurious; (ii) incorrect boundary conditions or oversimplified models were used when simulating flow, resulting in erroneous maps of WSS metrics in some or all of the studies; (iii) atherosclerosis is triggered by different haemodynamic factors in different vascular beds. The latter possibility, which we have termed the Comfort Zone hypothesis [51], could arise if endothelial cells become activated when any of a number of fluid- or solid-mechanical stresses falls outside the range that can be accommodated by homeostatic mechanisms. Some arterial locations and species might generate more extreme multidirectional flow, for example. Different fundamental properties of endothelial cells, perhaps arising from their various embryological origins [52], could also be a factor.

### Limitations

Disease prevalence and WSS metrics were obtained in different vessels. That was necessitated by the aim of investigating the initial stages of atherosclerosis. Early fatty streaks can only be identified postmortem, whilst assessing flow in time-varying geometries requires in vivo imaging. Both studies used a large enough number of vessels that rare geometries or lesion patterns would not have substantially biased the results, and men and women and a range of ages were included in both. Nevertheless, there may have been differences between the two populations. The data were collected in different countries at different times and the mean age was greater for the flow study.

Only the lesion maps were affected by postmortem shrinkage. Since both the PDAY study and our flow calculations examined the full circumference of the vessel, we compensated for shrinkage in diameter simply by adjusting the width of the WSS map. That was not possible for shrinkage in length. Instead, we stretched the lesion maps lengthwise, from 6 to 8 cm (33%). The degree of stretch was based on the shrinkage data of van den Broek et al. [40], but other values have been published [17]. We therefore also examined the effect of leaving the 6-cm pathology specimen unstretched, and of stretching it up to 10 cm in 1-cm increments. The correlation between lesion prevalence and RRT was maximal at the 8 cm we used in the main study but did not fall by more than 27% at other stretches (6 cm, ρ = 0.52; 7 cm, ρ = 0.63; 8 cm, ρ = 0.71; 9 cm, ρ = 0.68; 10 cm, ρ = 0.65). Changes in correlation coefficient as a function of stretch for other WSS metrics are shown in Supplementary Material Table S5; different stretches did not produce any large-scale changes in the pattern of coefficients across metrics.

The validity of the flow computations depends chiefly on the accuracy of the boundary conditions. Blood was modelled as a continuum with Newtonian rheology. That is a widely used approximation. The continuum assumption should have only small effects in vessels of this size, and we have previously shown that the same is true for the Newtonian approximation [26].

Perhaps more importantly, although the geometry tapered in a physiological manner, the cross section at each lengthwise location was approximated by an ellipse that was kept constant during the cardiac cycle. We did use inflow waveforms obtained in vivo—that is, under conditions where cross sections will have varied—so at least the influence of time-varying geometry on flow waveform was taken into account.

Flow waveforms could not be recorded in the subjects from whom RCA geometries were derived as Doppler flow wires approved for clinical use are no longer commercially available. The waveforms we obtained from previous studies were all made in patients undergoing routine invasive clinical investigation, like the subjects of the present study, by placing a combined pressure and velocity probe (ComboWire; Philips Volcano) in the proximal RCA.

The waveform in the side branches was assumed to have the same waveform as, and to be in phase with, flow in the RCA itself; measurement of branch waveforms is not technically feasible at present.

Velocities were obtained from the envelope of the Doppler ultrasound traces, which gives the maximum value occurring across the sample volume at each timepoint. By imposing these values at the centreline of the parabolic inlet velocity profile, we effectively assumed that the cross-sectionally averaged velocity in the simulations was half the measured value. That may have been inaccurate if the velocity profile at the measurement site was highly skewed; it would have led to an inappropriate scaling of true velocity magnitudes.

The cylindrical flow extension at the inlet is equivalent to assuming fully developed pulsatile flow at the entrance of the RCA. The physiological inlet profile will be different as a result of the complex flow in the Sinus of Valsalva. To investigate the possibility that this degraded the correlations between WSS metrics and lesion prevalence, we recomputed the correlations after removing the first part of the maps. Excluding the proximal 2 mm increased the correlation with RRT only modestly, from 0.71 to 0.75, and removing the proximal 3 mm increased it by a further small increment, to 0.76.

Twisting could not be imaged and was therefore not directly taken into account. Cyclical twisting of a regular cylinder combined with purely axial flow would give rise to non-zero OSI, transWSS and CFI; effects of this type may have been missed. However, twisting of a curved tube would have given rise to torsion (out-of-plane bending), and that was imaged and included in the model.

### Conclusions

Lesion prevalence in the human RCA had a low spatial correlation with TAWSS, the OSI and transWSS (ρ = − 0.23 to  + 0.25), and correlated only slightly better with the CFI and ECAP (ρ = 0.38–0.42). Correlations were greater with the ECAP when it was based on the CFI rather than OSI (ρ = 0.55), and were even higher with the RRT whether it was based on the OSI or CFI (ρ = 0.69–0.71). The OSI generally reflected multidirectional rather than forward-and-backward flow, presumably because inflow waveforms were forward directed throughout the cardiac cycle. The correlations are thus consistent with a combination of low time average and highly multidirectional WSS accounting for around half the spatial variation in the prevalence of fatty streaks, even though correlations with the two individual components were unimpressive.

## Supplementary Information

Below is the link to the electronic supplementary material.

Supplementary file1 (DOCX 5825 KB)
