# Supplementary material for: Relative Residence Time Can Account for Half of the Anatomical Variation in Fatty Streak Prevalence Within the Right Coronary Artery
Source: Ann Biomed Eng. 2024 Sep 17;53(1):144–57. doi: 10.1007/s10439-024-03607-9 (PMC11782302; doi:10.1007/s10439-024-03607-9)
Supplement: Supplementary file 1 — Supplementary file1 (DOCX 5825 KB) [file 10439_2024_3607_MOESM1_ESM.docx]

**Supplementary Material**

**Relative Residence Time can account for half of the anatomical variation in fatty streak prevalence within the right coronary artery**

Pratik Kandangwa^1,2^, Kevin Cheng^3^ , Miten Patel^3,4^, Spencer J. Sherwin^2^, Ranil de Silva^3,4^ and Peter D. Weinberg^1,*^

^1^ Department of Bioengineering, Imperial College London, London SW7 2AZ, UK

^2^ Department of Aeronautics, Imperial College London, London SW7 2AZ, UK

^3^ National Heart and Lung Institute, Imperial College London, London SW3 6LY, UK

^4^ Royal Brompton Hospital, Sydney Street, London SW3 6NP, UK


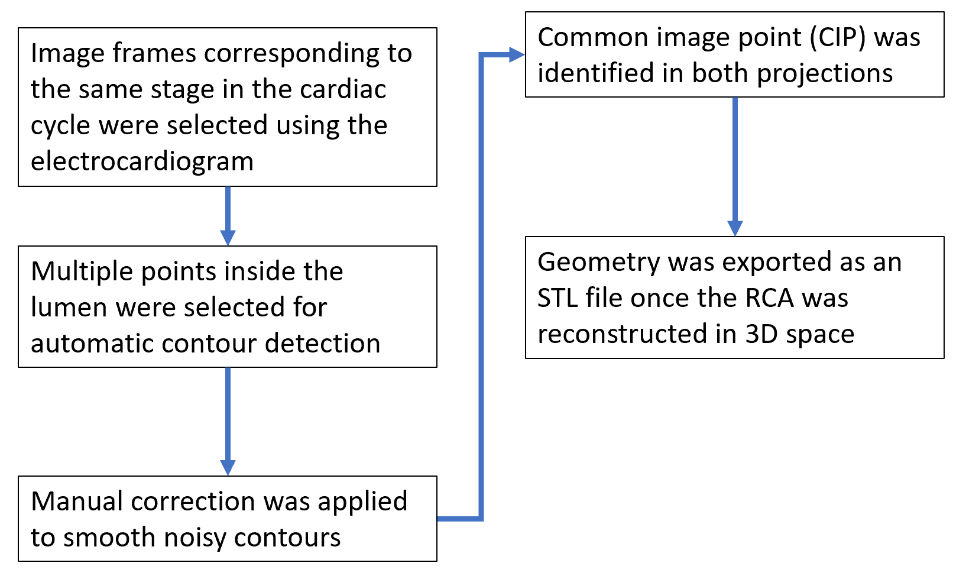


**Figure S1** workflow for the reconstruction of coronary geometries

**Table S1** WSS metrics were calculated according to the following equations where $\vec{\tau_{w}}$ is the instantaneous WSS vector, $\vec{n}$ is the surface normal and T is the period of the cardiac cycle:


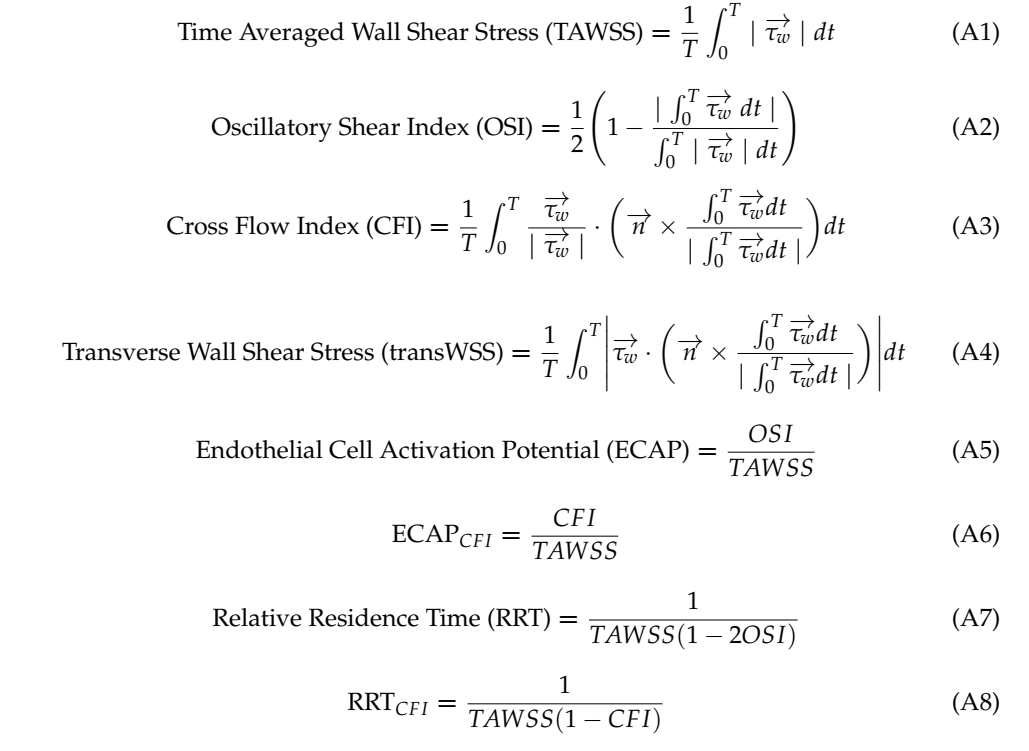


| Patient ID | Sex | Inlet hydraulic diameter (mm) | Time period (s) | Length  imaged (cm) | Number of branches | Womersley number (α) | Reynolds number (Re) |
| --- | --- | --- | --- | --- | --- | --- | --- |
| 001 | F | 3.8 | 1 | 8.8 | 3 | 2.4 | 303 |
| 009 | F | 3.25 | 0.94 | 10.2 | 3 | 2.1 | 259 |
| 010 | M | 2.85 | 0.83 | 8.4 | 3 | 2 | 227 |
| 011 | F | 3.33 | 0.87 | 8.3 | 3 | 2.3 | 266 |
| 012 | F | 3.68 | 0.88 | 11 | 4 | 2.5 | 294 |
| 014 | M | 3.12 | 0.79 | 8.9 | 2 | 2.2 | 249 |
| 019 | F | 3.91 | 0.86 | 10.9 | 5 | 2.7 | 312 |
| 023 | M | 2.7 | 0.98 | 8 | 2 | 1.7 | 215 |
| 026 | M | 3.66 | 0.7 | 9.8 | 4 | 2.8 | 292 |
| 029 | M | 4.2 | 0.9 | 10.3 | 4 | 2.7 | 335 |

**Table S2** Subject geometrical features and flow parameters

|  | Mean (BC1) | Mean (BC2) | Mean (BC3) | SD  (BC1) | SD  (BC2) | SD  (BC3) |
| --- | --- | --- | --- | --- | --- | --- |
| TAWSS | 1.3 | 3.3 | 2.2 | 0.41 | 1.1 | 0.71 |
| OSI | 0.024 | 0.0086 | 0.011 | 0.007 | 0.0058 | 0.0056 |
| CFI | 0.16 | 0.13 | 0.14 | 0.048 | 0.042 | 0.042 |
| transWSS | 0.15 | 0.41 | 0.26 | 0.051 | 0.15 | 0.093 |
| ECAP | 0.021 | 0.0033 | 0.0059 | 0.011 | 0.0034 | 0.0052 |
| ECAP_CFI_ | 0.15 | 0.047 | 0.071 | 0.088 | 0.03 | 0.044 |
| RRT | 0.93 | 0.35 | 0.51 | 0.34 | 0.14 | 0.20 |
| RRT_CFI_ | 1.1 | 0.40 | 0.59 | 0.43 | 0.17 | 0.24 |

**Table S3** Mean and standard deviation of the values for each WSS metric shown in Figure 2

|  | Mean (Branched) | Mean (Branchless) | SD  (Branched) | SD  (Branchless) |
| --- | --- | --- | --- | --- |
| TAWSS | 2.2 | 3.3 | 0.44 | 1.1 |
| OSI | 0.011 | 0.0086 | 0.0066 | 0.0058 |
| CFI | 0.13 | 0.13 | 0.042 | 0.042 |
| transWSS | 0.26 | 0.41 | 0.096 | 0.15 |
| ECAP | 0.0051 | 0.0033 | 0.0039 | 0.0034 |
| ECAP_CFI_ | 0.062 | 0.047 | 0.028 | 0.03 |
| RRT | 0.48 | 0.35 | 0.13 | 0.14 |
| RRT_CFI_ | 0.54 | 0.40 | 0.15 | 0.17 |

**Table S4** Mean and standard deviation of the values for each WSS metric shown in Figure 3


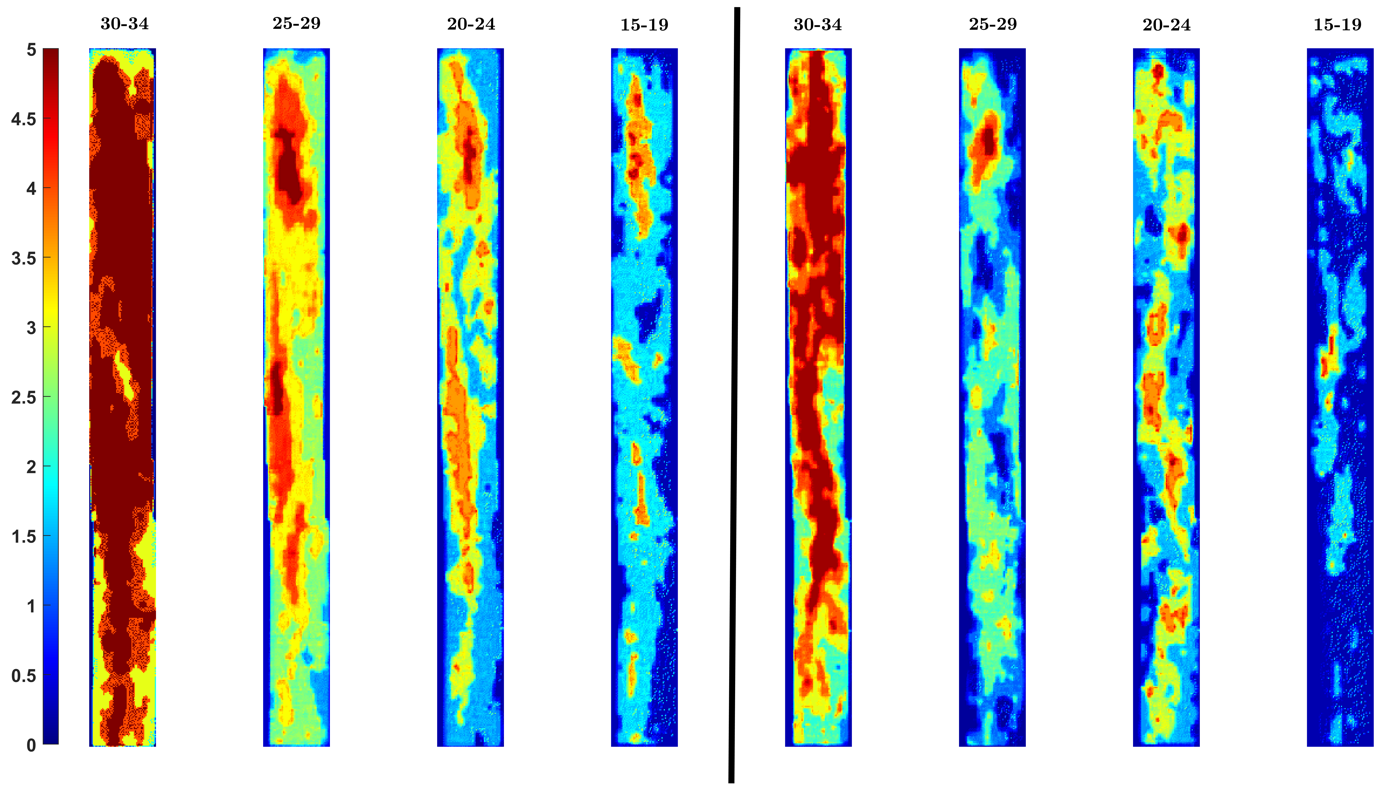


**Figure S2** Maps showing fatty streak prevalence averaged for four individual age ranges (values in years), for male (left of black line) and female (right of black line) subjects of the PDAY study separately. Colour coding shows lesion prevalence in arbitrary units. Mean RCA flow from top to bottom of the map.


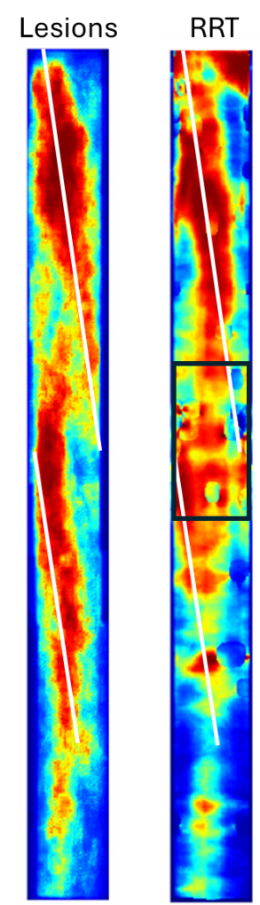


**Figure S3** Maps showing lesion prevalence averaged across age groups (Left) and relative residence time averaged across subjects (RRT; Right). Superimposed white lines show the putatively helical pattern in both cases. (The vessels were physically or virtually opened along their outer aspect for both maps.) There is additionally a spottiness in the RRT map in the region indicated by the black box, where major branches tend to occur but with a precise location that varies from subject to subject. The effects of those branches will be less smoothed in the CFD maps than the lesion maps, due to the much smaller sample size. Colour coding shows lesion prevalence and RRT as described for Figures 4a and 4b. Mean RCA flow from top to bottom of the map.


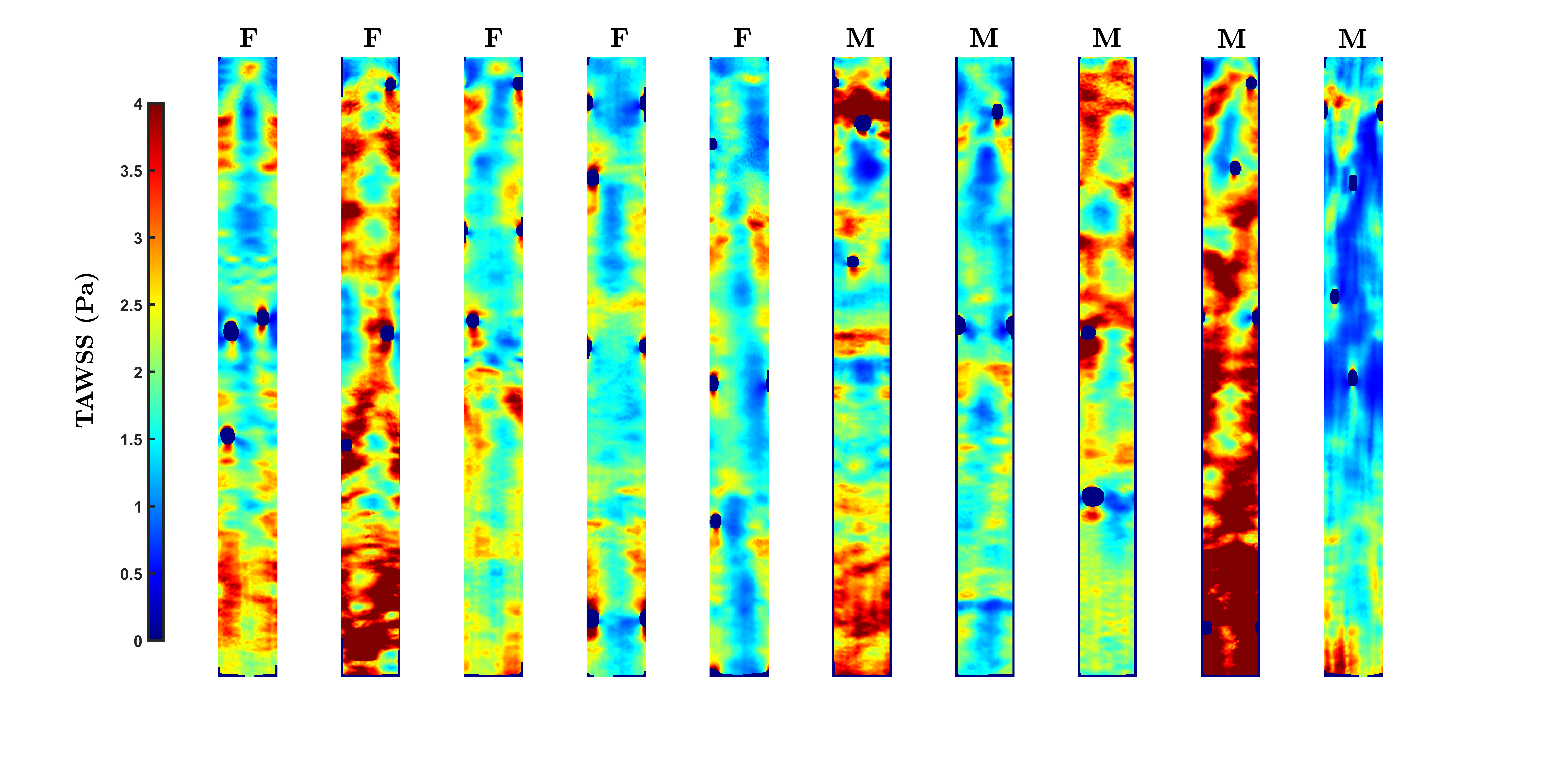


**Figure S4** Maps of TAWSS for all 10 subjects (F-Female and M-Male, mean RCA flow from top to bottom of the map)


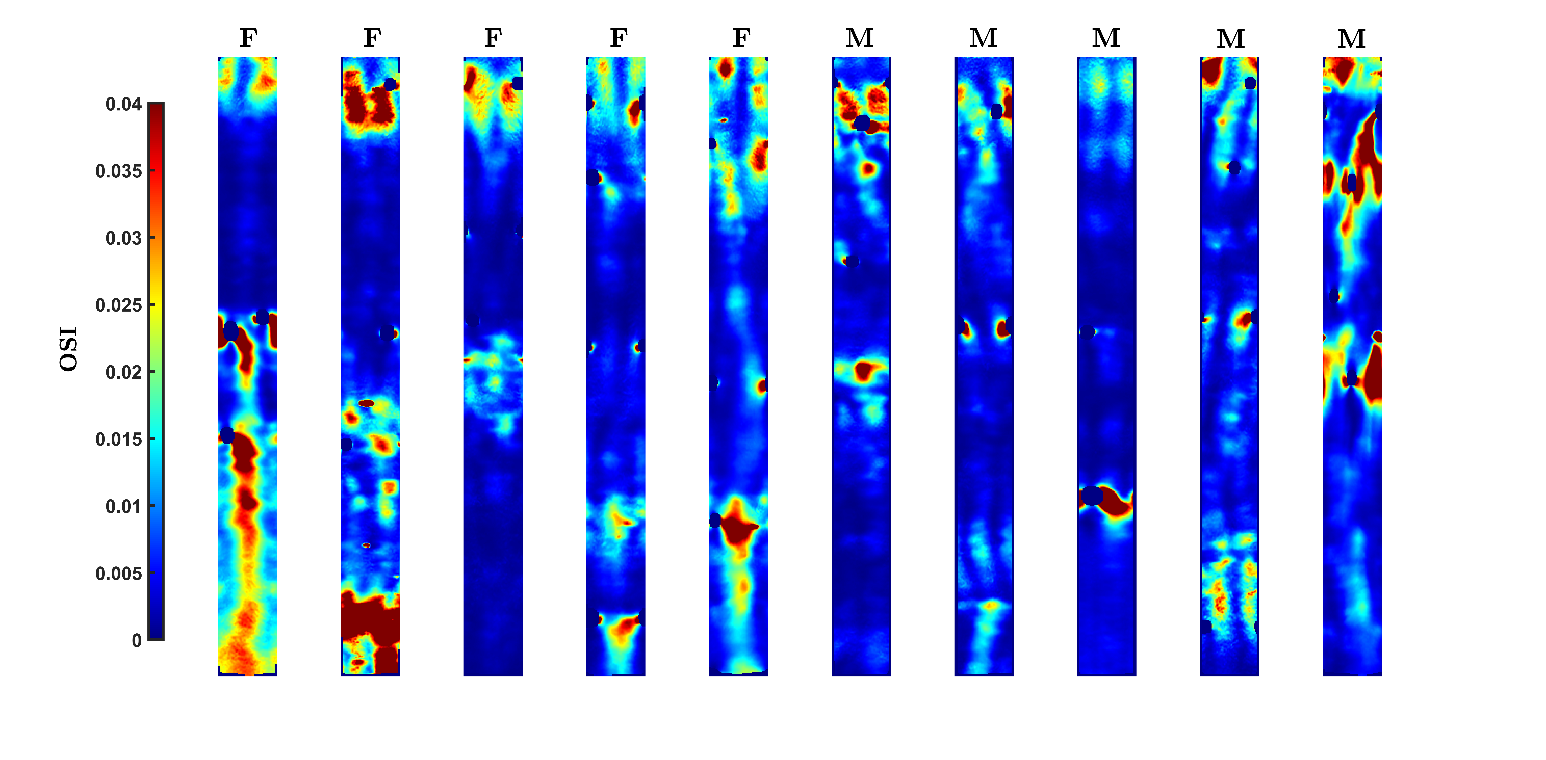


**Figure S5** Maps of OSI for all 10 subjects (F-Female and M-Male, mean RCA flow from top to bottom of the map)

*
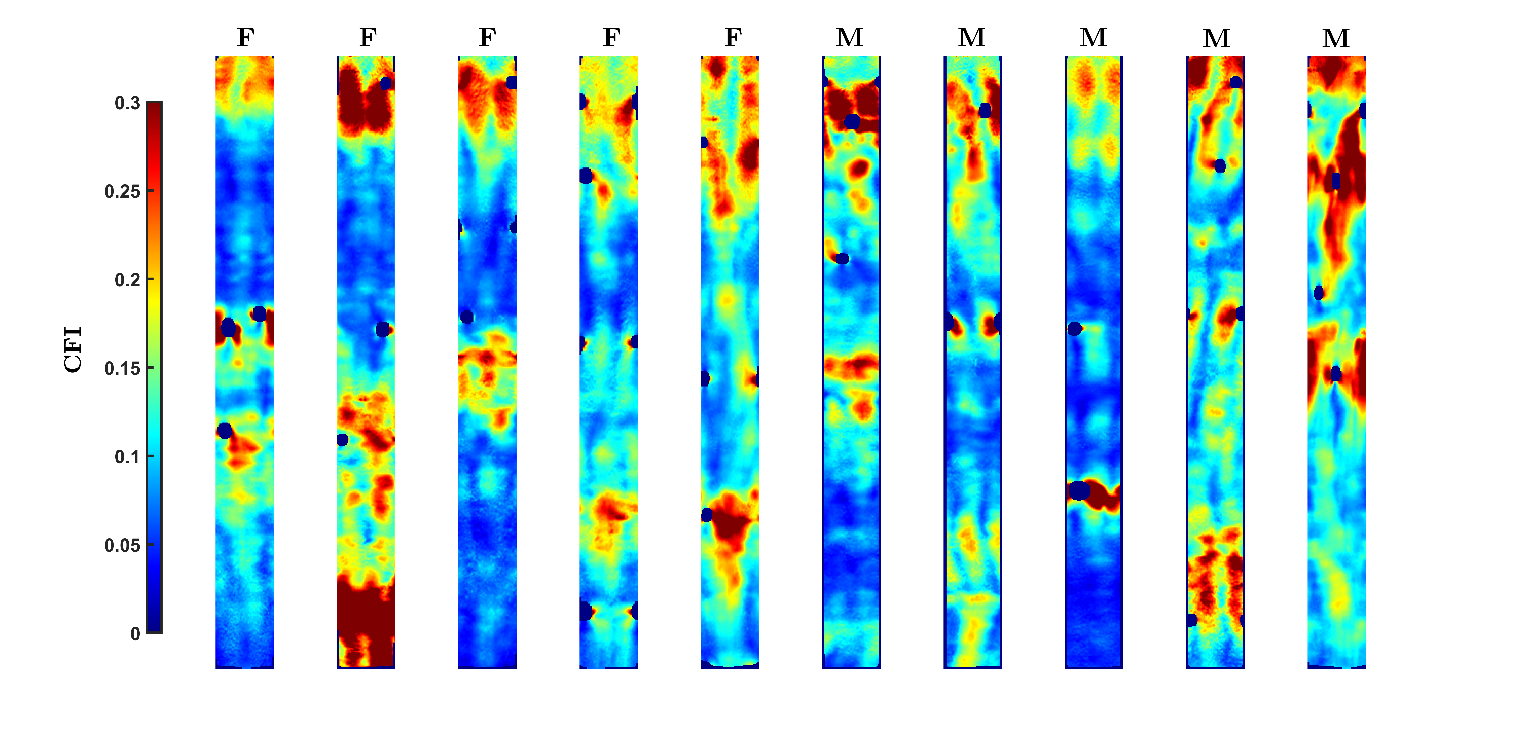
*

**Figure S6** Maps of CFI for all 10 subjects (F-Female and M-Male, mean RCA flow from top to bottom of the map)

***
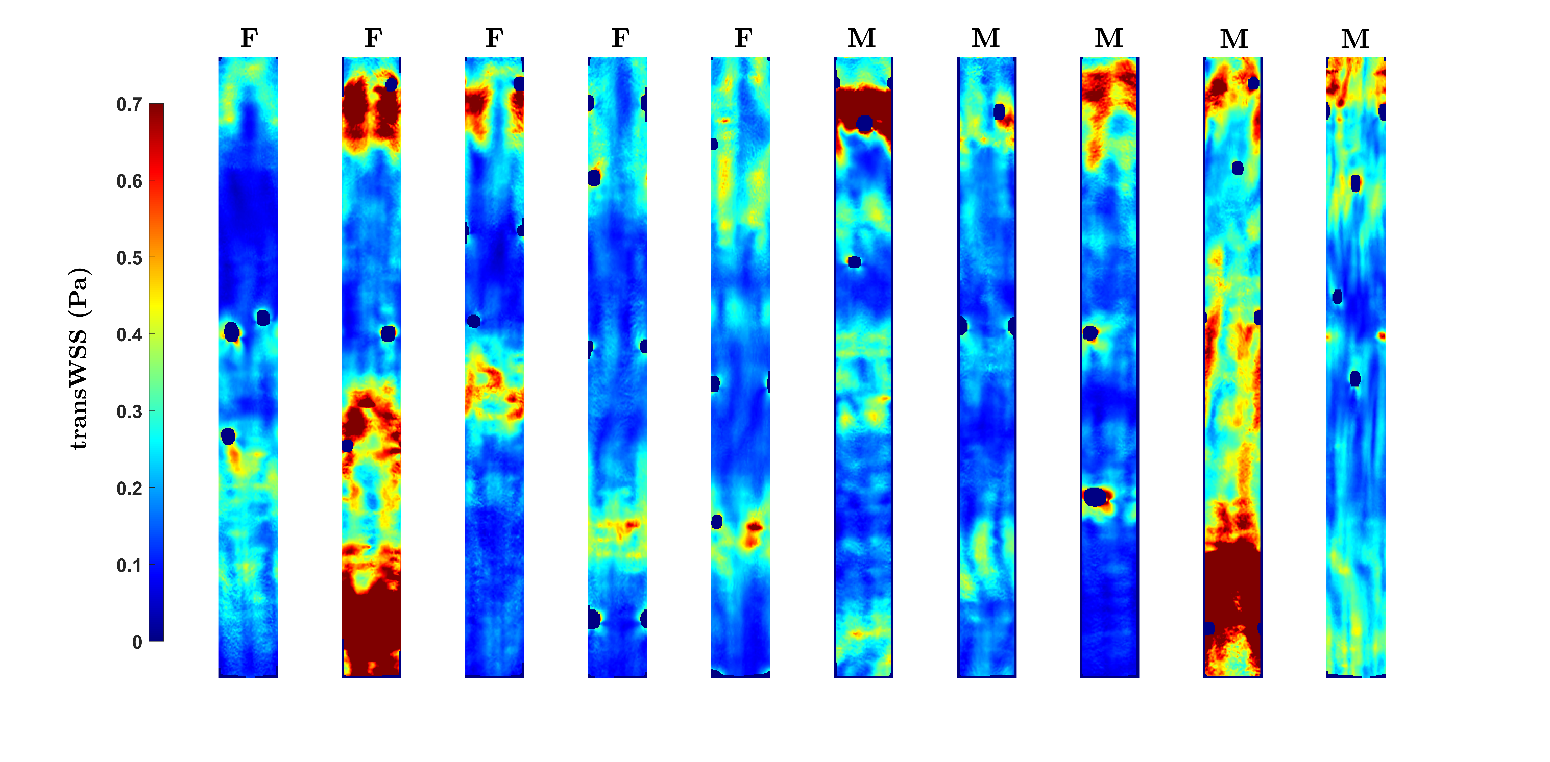
***

**Figure S7** Maps of transWSS for all 10 subjects (F-Female and M-Male, mean RCA flow from top to bottom of the map)


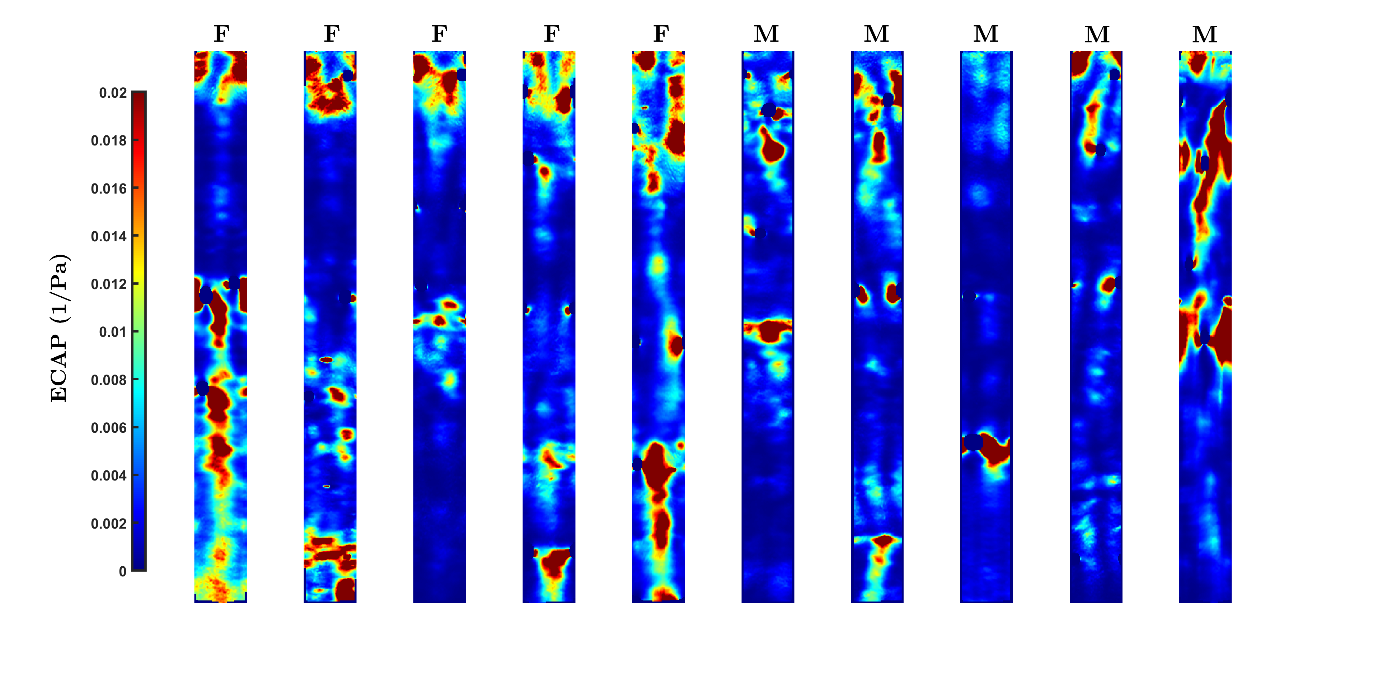


**Figure S8** Maps of ECAP all 10 subjects (F-Female and M-Male, mean RCA flow from top to bottom of the map)


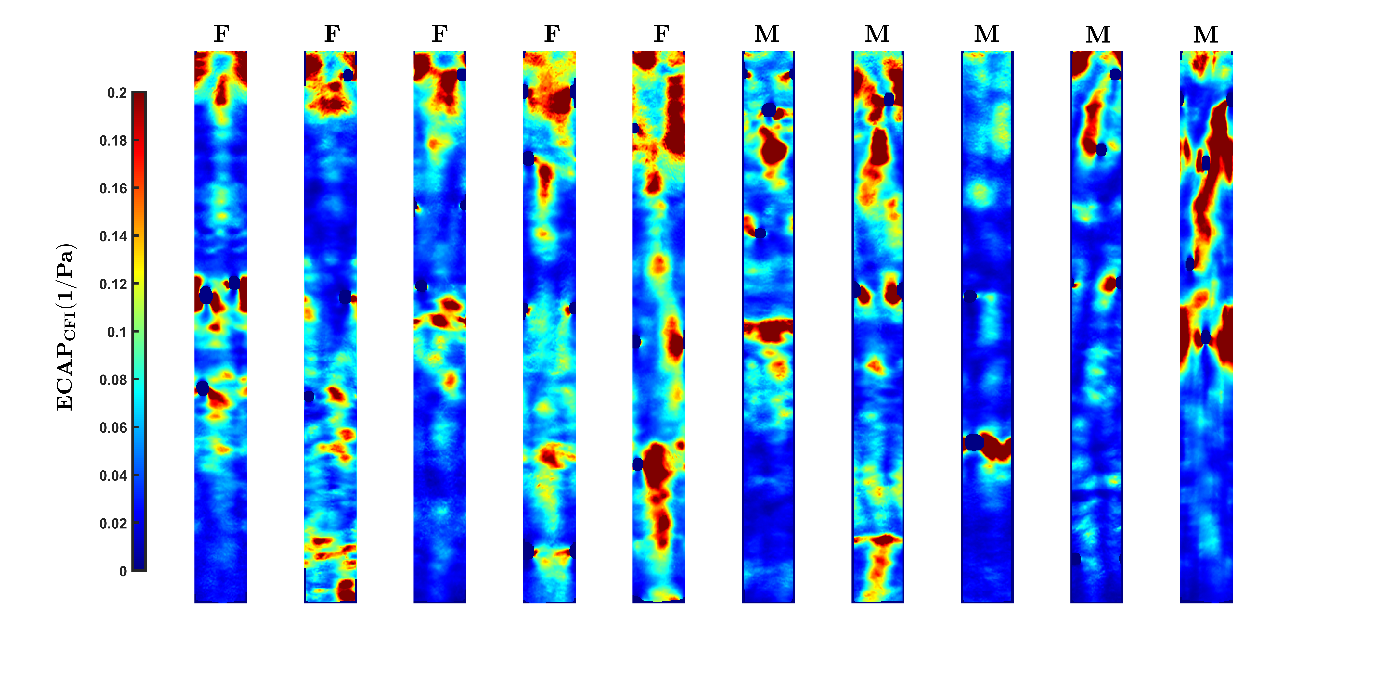


**Figure S9** Maps of ECAP_CFI_ all 10 subjects (F-Female and M-Male, mean RCA flow from top to bottom of the map)


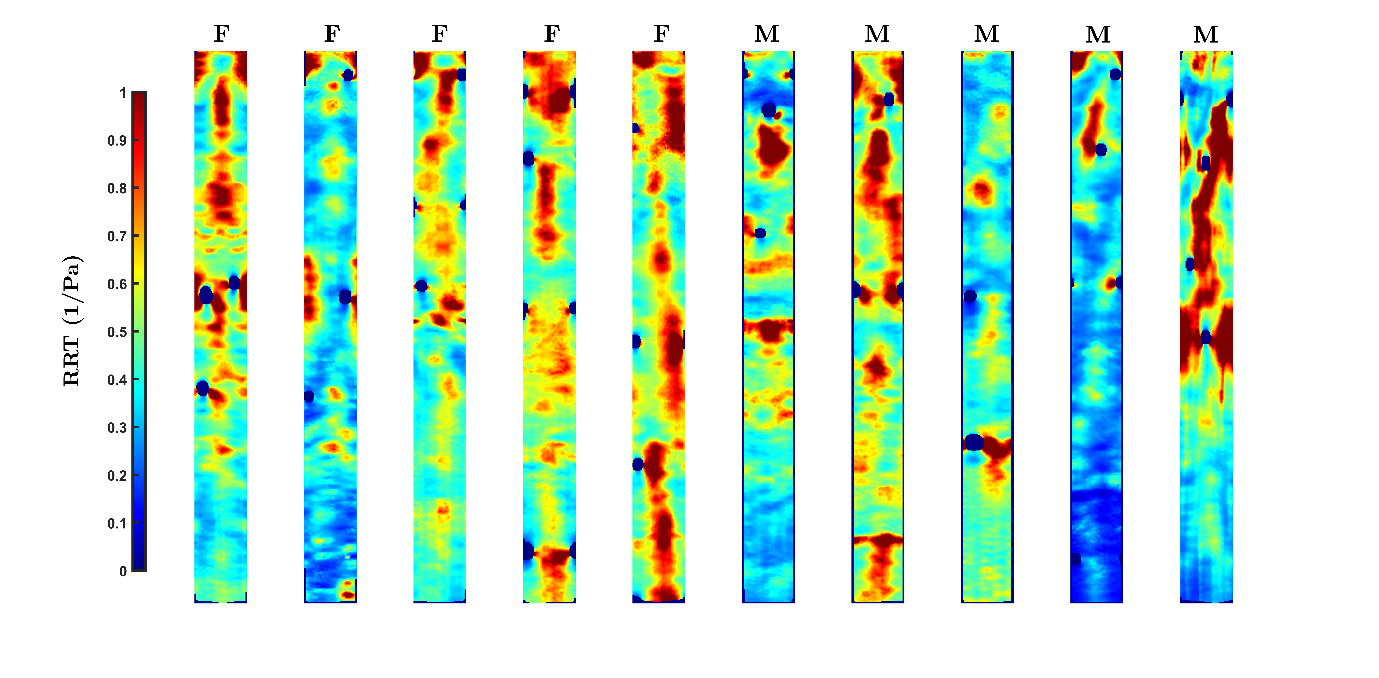


**Figure S10** Maps of RRT all 10 subjects (F-Female and M-Male, mean RCA flow from top to bottom of the map)


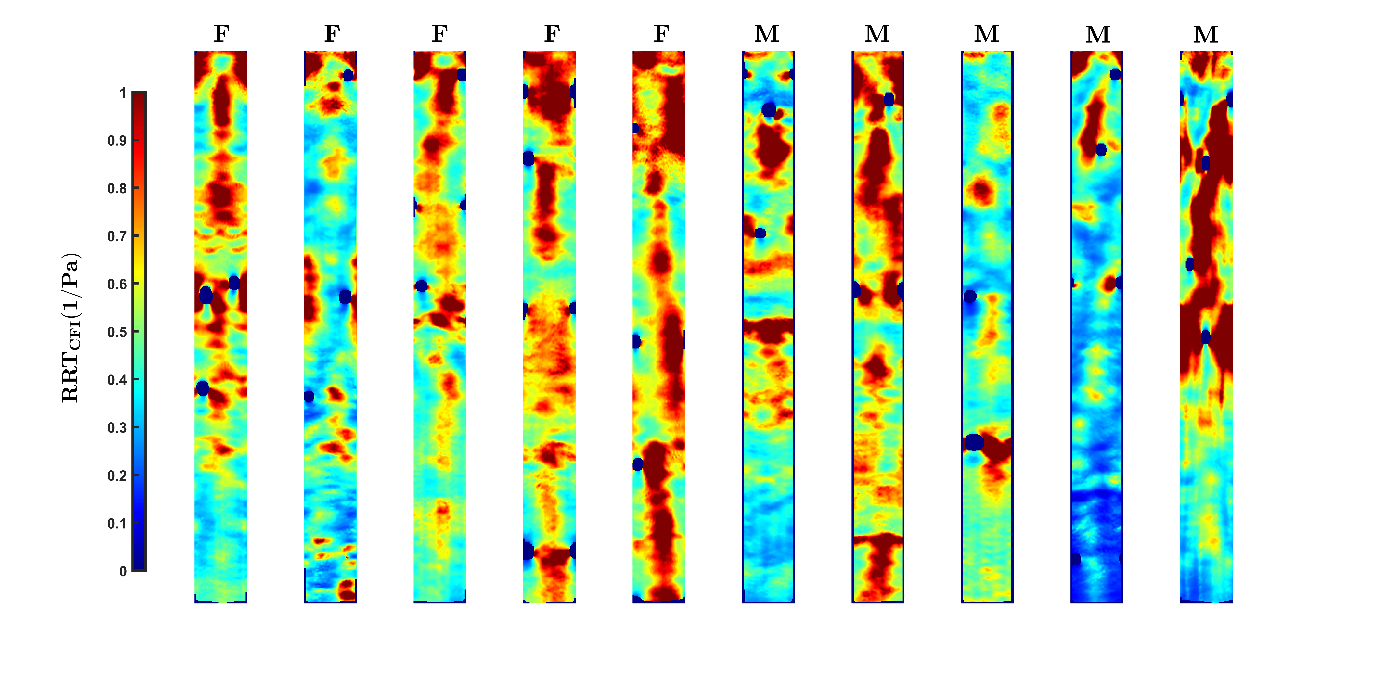


**Figure S11** Maps of RRT_CFI_ all 10 subjects (F-Female and M-Male, mean RCA flow from top to bottom of the map)

*
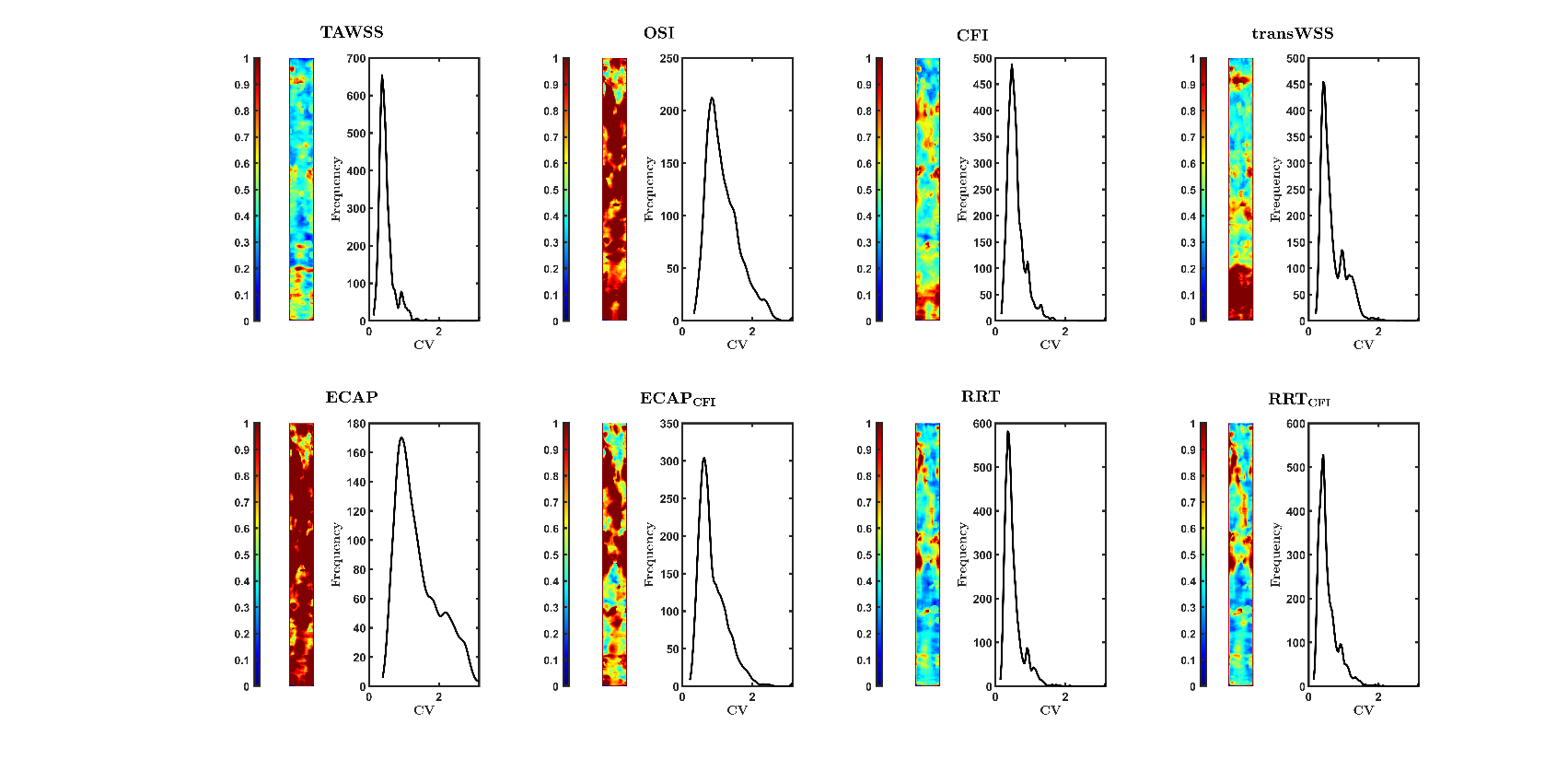
***Figure S12** Maps and frequency distributions of the Coefficient of Variation (CV; mean/standard deviation) for WSS metrics computed with inflow waveform BC2. Mean RCA flow is from top to bottom of the map.

|  | Length to which postmortem data were stretched | | | | |
| --- | --- | --- | --- | --- | --- |
|  | 6cm | 7cm | 8cm | 9cm | 10cm |
| WSS metric |  |  |  |  |  |
| TAWSS | -0.098 | -0.21 | -0.23 | -0.23 | -0.23 |
| OSI | 0.17 | 0.25 | 0.25 | 0.25 | 0.25 |
| CFI | 0.31 | 0.35 | 0.42 | 0.41 | 0.41 |
| transWSS | 0.0098 | 0.0053 | -0.028 | -0.035 | -0.035 |
| ECAP | 0.17 | 0.31 | 0.38 | 0.38 | 0.38 |
| ECAP_CFI_ | 0.31 | 0.44 | 0.55 | 0.53 | 0.53 |
| RRT | 0.52 | 0.63 | 0.71 | 0.68 | 0.65 |
| RRT_CFI_ | 0.49 | 0.59 | 0.69 | 0.65 | 0.65 |

**Table S5** Spearman’s rank correlation coefficient (ρ) obtained by pointwise comparison of maps of eight WSS metrics with the average map of lesion prevalence at its original 6 cm length or stretched to 7 - 10 cm

*Appendix A*

Bootstrapping method:

1. The original sample of 10 individual maps for each WSS metric was resampled with replacement. That is, one map from the original sample was chosen and then put back, then a second was chosen and put back, and so on until ten had been selected; because of the replacement, some individual maps from the original sample might be chosen more than once and others might not get chosen at all, leading to a different average map.
2. 10,000 average maps were created in this way.
3. Spearman’s rank correlation coefficient was computed between each bootstrapped average map and the disease prevalence map to generate a distribution of values of ρ.
4. To obtain the 95% CI, the 2.5th and 97.5th percentiles were taken as the lower and upper bounds, respectively.

*Appendix B*

We examined potential determinants of the two components of high RRT – low TAWSS and highly multidirectional flow – to further understand what might trigger the development of atherosclerosis.

TAWSS is related to volumetric flow rate and vessel radius. Figure S13 shows TAWSS plotted against radius for different points along two RCAs. One of the RCAs shows a strong inverse correlation while the other shows a weaker, albeit still substantial, inverse correlation. Both RCAs had four branches, but the branches were larger in the one showing the weaker inverse correlation, resulting in 50% rather than 30% of the inlet flow leaving through them. We conclude that axial regions of low or high TAWSS do depend on taper but cannot be entirely predicted by looking at the local radius; effects of branches on flow rate must additionally be taken into account.

**
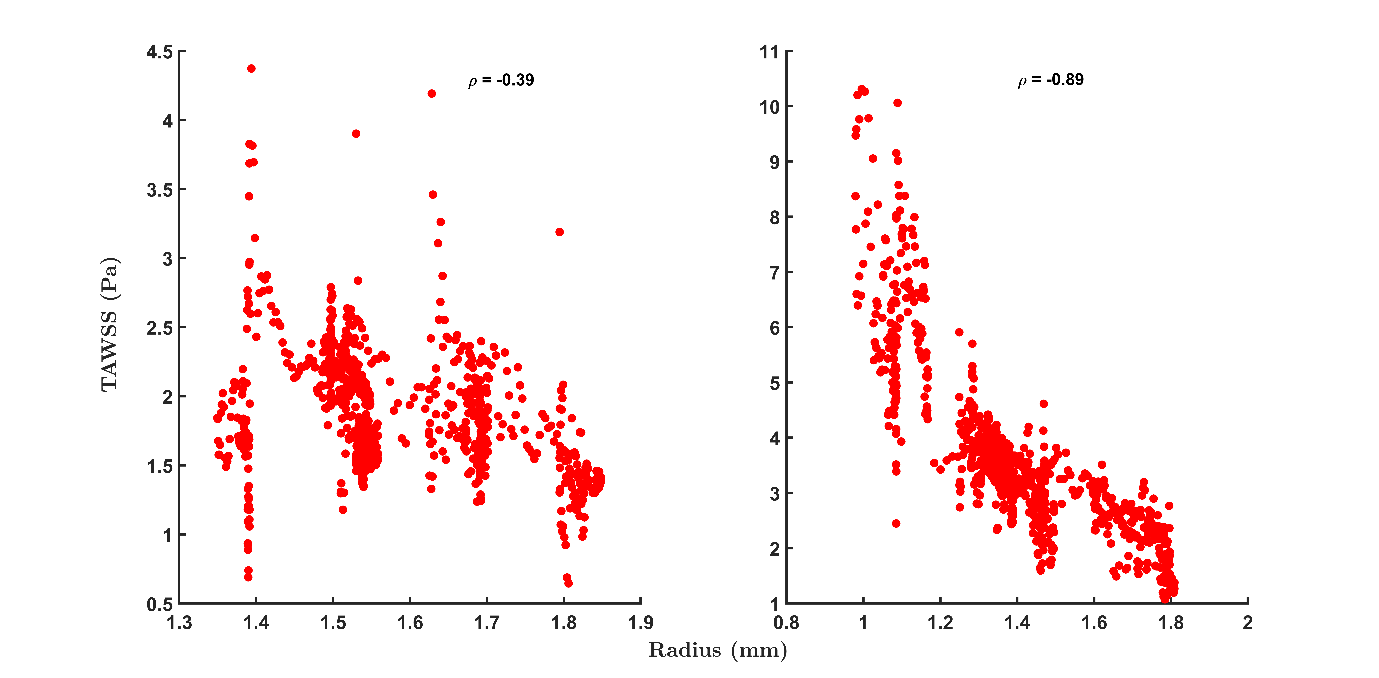
**

**Figure S13** Relation between TAWSS and radius for two different RCAs.

Wall motion is characterised by a combination of translation and changes in curvature and torsion. Figure S14 shows how these three variables – each assessed every 0.5mm along the centreline, summed over the cardiac cycle and normalised to have a maximum value of 1 – vary along the length of one RCA. It is clear from this example that excursions of the three do not necessarily coincide. Figure S15 shows scatter plots of the three components of motion against circumferentially averaged CFI for all 10 subjects. Spearman’s rank correlation coefficients, summarised in Table S6, show a moderate positive correlation of CFI with change in curvature (0.51) and weak negative correlations for both translation and change in torsion (-0.18 and -0.21, respectively).


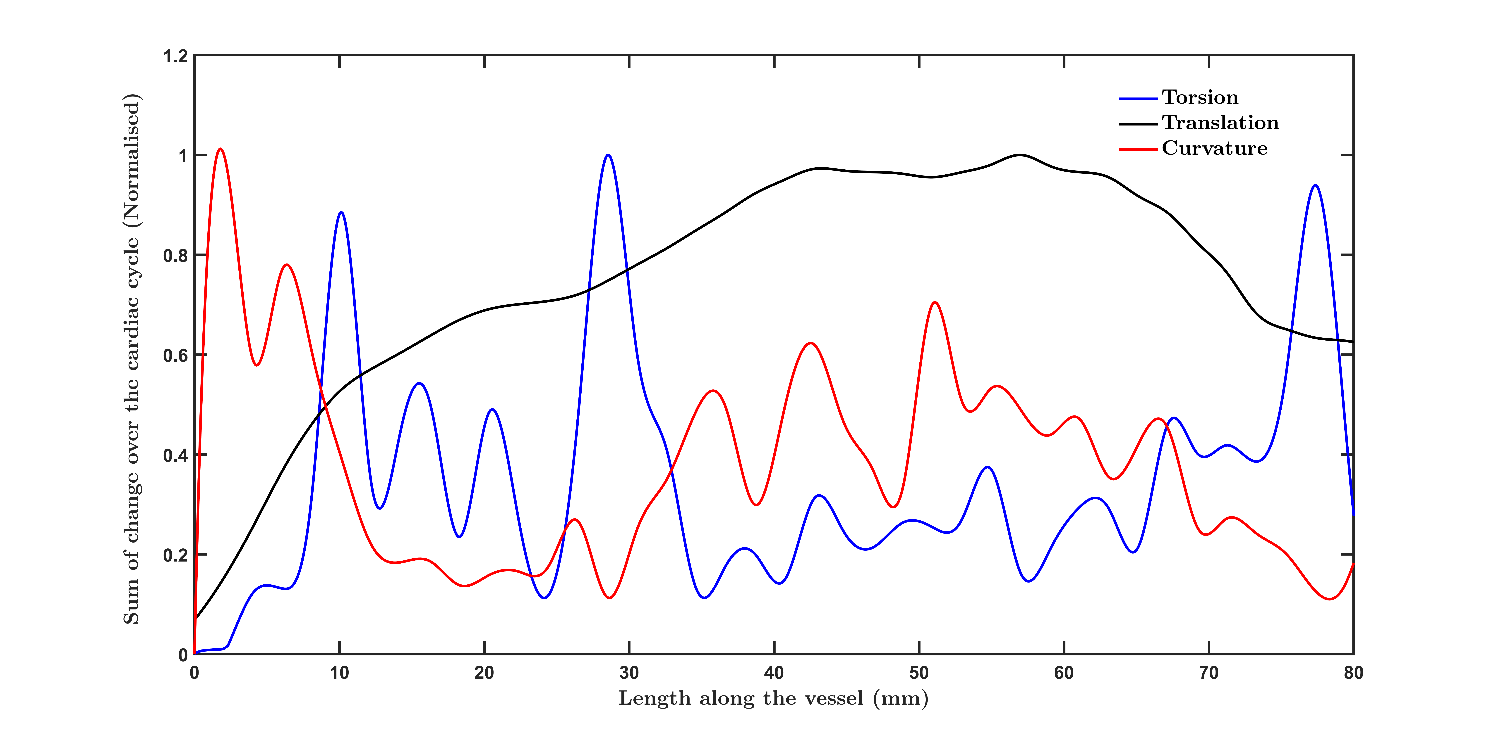


**Figure S14** Normalised sum of translation and of the change in curvature and torsion over the cardiac cycle as a function of distance from the origin of an RCA


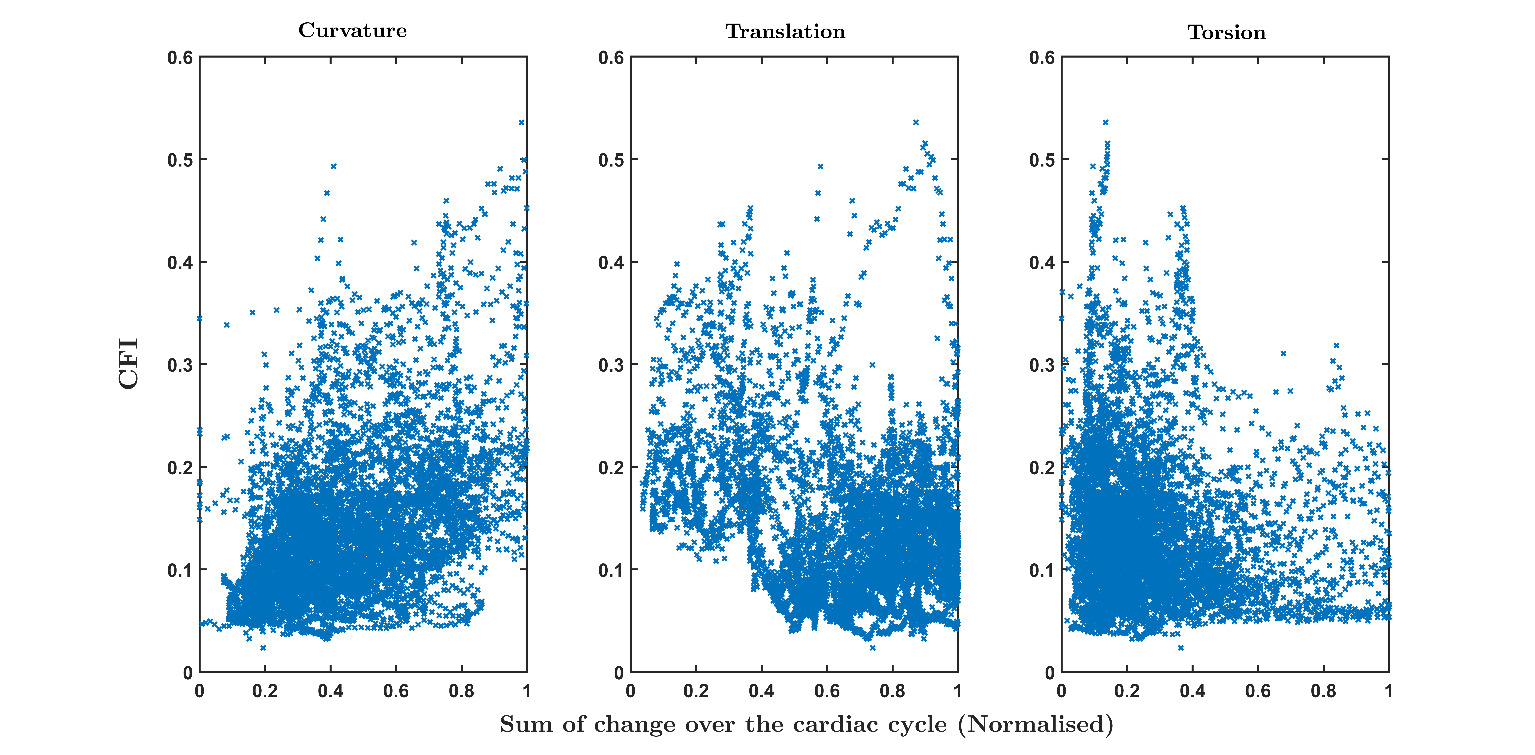


**Figure S15** Scatter plot of CFI against the sum of changes over the cardiac cycle in the three constituents of RCA motion

| Type of motion | ρ |
| --- | --- |
| Curvature | 0.51 |
| Translation | -0.18 |
| Torsion | -0.21 |

**Table S6** Spearman’s rank correlation coefficient (ρ) between CFI and changes in the three constituents of motion, summed over the cardiac cycle, across 10 subjects
